# Dysregulation of Non-Coding RNAs: Roles of miRNAs and lncRNAs in the Pathogenesis of Multiple Myeloma

**DOI:** 10.3390/ncrna9060068

**Published:** 2023-11-03

**Authors:** Nor Hayati Ismail, Ali Mussa, Mutaz Jamal Al-Khreisat, Shafini Mohamed Yusoff, Azlan Husin, Hamid Ali Nagi Al-Jamal, Muhammad Farid Johan, Md Asiful Islam

**Affiliations:** 1 Department of Haematology, School of Medical Sciences, Universiti Sains Malaysia, Kubang Kerian 16150, Kelantan, Malaysia; 2 Department of Biology, Faculty of Education, Omdurman Islamic University, Omdurman 11111, Sudan; 3 Center for Global Health Research, Saveetha Medical College and Hospitals, Saveetha Institute of Medical and Technical Sciences, Saveetha University, Chennai 600077, Tamil Nadu, India; 4 Department of Internal Medicine, School of Medical Sciences, Universiti Sains Malaysia, Kubang Kerian 16150, Kelantan, Malaysia; 5 School of Biomedicine, Faculty of Health Sciences, Universiti Sultan Zainal Abidin (UniSZA), Kuala Nerus 21300, Terengganu, Malaysia; 6 WHO Collaborating Centre for Global Women’s Health, Institute of Metabolism and Systems Research, College of Medical and Dental Sciences, University of Birmingham, Birmingham B15 2TT, UK

**Keywords:** multiple myeloma, pathogenesis, dysregulation, non-coding RNAs, microRNAs, long non-coding RNAs

## Abstract

The dysregulation of non-coding RNAs (ncRNAs), specifically microRNAs (miRNAs) and long non-coding RNAs (lncRNAs), leads to the development and advancement of multiple myeloma (MM). miRNAs, in particular, are paramount in post-transcriptional gene regulation, promoting mRNA degradation and translational inhibition. As a result, miRNAs can serve as oncogenes or tumor suppressors depending on the target genes. In MM, miRNA disruption could result in abnormal gene expression responsible for cell growth, apoptosis, and other biological processes pertinent to cancer development. The dysregulated miRNAs inhibit the activity of tumor suppressor genes, contributing to disease progression. Nonetheless, several miRNAs are downregulated in MM and have been identified as gene regulators implicated in extracellular matrix remodeling and cell adhesion. miRNA depletion potentially facilitates the tumor advancement and resistance of therapeutic drugs. Additionally, lncRNAs are key regulators of numerous cellular processes, such as gene expression, chromatin remodeling, protein trafficking, and recently linked MM development. The lncRNAs are uniquely expressed and influence gene expression that supports MM growth, in addition to facilitating cellular proliferation and viability via multiple molecular pathways. miRNA and lncRNA alterations potentially result in anomalous gene expression and interfere with the regular functioning of MM. Thus, this review aims to highlight the dysregulation of these ncRNAs, which engender novel therapeutic modalities for the treatment of MM.

## 1. Introduction

### 1.1. Clinical Aspects of Multiple Myeloma

Multiple myeloma (MM) is an incurable hematological malignancy caused by the aberrant proliferation of plasma cells in the bone marrow [1]. This disease causes bone marrow failure, which leads to anemia, low immunity, bone pain and fractures, hypercalcemia, and renal failure. These symptoms manifest from the aggregation of malignant plasma cells and their corresponding by-products within the bone marrow and other body parts [2]. The diagnostic evaluation entails hematological and urinary analyses, bone marrow biopsy, and imaging such as X-rays, magnetic resonance imaging (MRI), and computed tomography (CT) scans to assess the extent of bone involvement. Specific MM biomarkers, such as monoclonal proteins (M-proteins) and free light chains, are often employed in medical diagnosis and ongoing disease monitoring [3]. The staging of MM patients is determined based on the International Staging System (ISS) and the Revised International Staging System (R-ISS) [4]. Notably, MM prognosis exhibits considerable heterogeneity among individuals as the disease is influenced by a multitude of factors, including genetic aberrations, advanced age, and co-existing medical conditions. The presence of high-risk cytogenetic abnormalities, such as the occurrence of t (4;14) or del (17p), has been correlated with a less favorable prognosis [5].

### 1.2. Biology of MM

MM is characterized by a multitude of genetic modifications [6], for instance, chromosomal translocations in the immunoglobulin heavy chain (IgH) locus, namely t (11;14), t (4;14), and t (14;16) [7], and dysregulation of oncogenes such as CCND1, MMSET, and MAF [8]. Deletions and mutations in the genetic loci that encode tumor suppressor genes, such as TP53, are correlated with poor patient outcomes [9]. Another crucial area that could help researchers understand the multifaceted nature of MM is epigenetic modification, such as DNA methylation, histone modifications, and the involvement of non-coding RNAs, all of which assume a pivotal role in MM pathogenesis. These dysregulations potentially induce the silencing of tumor suppressor genes or the activation of oncogenes [10]. The MM cells exhibit a crucial interplay with the complex microenvironment of the bone marrow, exerting a profound influence on the disease progression. The presence of MM cells perturbs the physiological process of bone remodeling, giving rise to the development of bone lesions, fractures, and the manifestation of bone pain. The microenvironment additionally facilitates the sustenance of MM cell viability and the development of resistance against therapeutic agents [10].

### 1.3. Therapeutic Challenges in MM

MM exhibits significant heterogeneity in genetic and clinical characteristics, posing challenges for healthcare providers in making treatment decisions and necessitating personalized therapeutic strategies [5]. A rapid development in treatment resistance is also observed in MM cells, resulting in relapse and refractory phases in nearly all patients after treatment [11]. Resistance mechanisms in MM are derived through various processes, such as genomic instability, clonal evolution, and tumor microenvironment alterations [12]. This disease also contributes to bone disease complications among MM patients, adding to the complexity of addressing bone lesions and fractures [13]. As MM advances, patients also experience immunosuppression, rendering them vulnerable to infections [14] and posing challenges in administering immunomodulatory drugs and immunotherapies.

The MM treatment often requires elaborate treatment regimens comprising various combination therapies. These regimens typically include chemotherapy, immunomodulatory drugs, proteasome inhibitors, and monoclonal antibodies. Managing these treatment plans and the associated adverse effects is a major hurdle clinicians face [15]; thus, it is almost impossible to standardize the treatments for all MM patients. While it is true that MM patients may initially respond well to treatment, it is essential to note that at later stages, they may develop a level of resistance. As a result, MM remains one of the cancers with higher financial implications [15].

Non-coding RNAs (ncRNAs) are a heterogeneous class of RNA molecules that lack protein-coding capacity but are essential for numerous cellular and molecular functions, such as the regulation of genes, processing of RNA, and genome preservation [16]. The ncRNAs can be effectively categorized based on their respective lengths, which are demonstrated into two distinct groups: small RNAs, encompassing those with a length of less than 200 nucleotides, and long RNAs, comprising those exceeding the 200-nucleotide threshold. Alternatively, these non-coding RNAs can also be classified based on their functional roles, distinguishing between housekeeping RNAs responsible for essential cellular functions and regulatory RNAs, which exert control over various biological processes [17]. Most importantly, ncRNAs in MM exemplify epigenetic modifications.

The elucidation of ncRNAs as prominent constituents of epigenetics has significantly augmented the existing knowledge concerning the biological processes underlying MM. Nevertheless, this field of study is still in its infancy and requires further investigation, specifically in ncRNA dysregulation in MM pathogenesis. Therefore, this review focuses on the current understanding of ncRNAs dysregulation in MM development, particularly miRNAs and lncRNAs. This knowledge is essential for the advancement of targeted therapeutic approaches and, hopefully, a cure for MM in the near future.

### 1.4. Roles of ncRNAs in MM

The literature has reported that ncRNAs are significantly dysregulated in human cancer. In MM, the ncRNAs significantly modulate gene expression post-transcriptionally, influencing diverse cellular mechanisms. Dysregulation of ncRNAs can deregulate critical genes implicated in MM development [18]. ncRNAs are reportedly involved in cellular development, physiology, and the pathology of various human diseases, including MM [19]. Consequently, ncRNAs have been recognized as promising biomarkers and therapeutic targets in various cancers including MM. Profiling and sequencing studies have identified notable dysregulation of ncRNAs in MM and are frequently attributed to abnormal mechanisms of ncRNA biogenesis, such as amplification, deletion, and aberrant epigenetic or transcriptional regulation [20]. Understanding the precise mechanisms underlying the dysregulation of ncRNAs in MM can offer valuable insights into developing targeted therapeutic approaches. For instance, lncRNAs regulate gene expression by functioning as independent transcriptional units or untranslated regions (UTRs) of protein-coding genes, crucial for regulating mechanisms of gene expression [21]. Raimondi et al. have revealed that the dysregulation of many subclasses of ncRNAs plays a critical role in regulating key signaling pathways in the pathogenesis of multiple myeloma bone disease [22]. Furthermore, Butova et al. provided a comprehensive overview of the existing understanding of ncRNA, with a specific emphasis on lncRNAs, and their involvement in the development of MM [21]. This review specifically addresses the existing knowledge on the aberrant regulation of ncRNAs, specifically both on miRNAs and lncRNAs, in the progression of MM. Acquiring this insight is crucial for the progress of specific therapy strategies and, optimistically, the development of a therapy for MM in the approaching future.

## 2. Non-Coding RNAs

The majority of the human genome (98.5%) is non-coding, functioning as a regulator of cellular physiology systems and pathological molecular change [21]. Breakthroughs in high-throughput sequencing and bioinformatics have put the spotlight on ncRNAs, which were traditionally considered trash RNAs. The ncRNAs are a diverse class of RNA molecules that do not code for proteins and are distinguished by their size, origin, biogenesis, and biological functions [23]. The ncRNAs participate in various intricate molecular processes, such as gene expression regulation, epigenetic modifications, and cellular signaling [24]. Highly expressed housekeeping ncRNAs offer new ways to treat cancer [25]. These ncRNAs have been described as key parts in epigenetic, transcriptional, and post-transcriptional gene control that dysregulates cellular processes [26,27,28]. Moreover, most ncRNAs can regulate gene expression without altering the DNA sequence, making them ideal targets for disease treatments. Furthermore, ncRNAs can act as tumor suppressors or oncogenic drivers and have been linked to numerous forms of cancer [29].

### 2.1. microRNAs (miRNAs)

**Figure 1:**
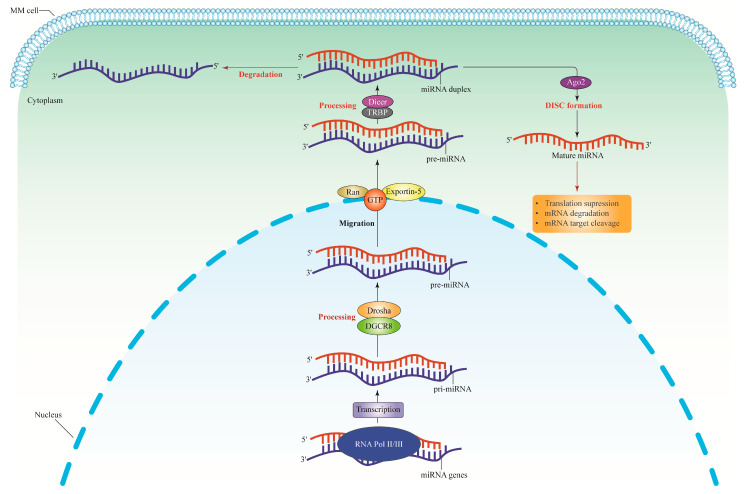
The miRNA biogenesis pathway. The miRNA genes are transcribed by RNA polymerase II to synthesize the pi-miRNAs. The pri-miRNAs are cleaved by Drosha and DGCR8 complex or microprocessor complex to produce pre-miRNAs which are approximately 65 nucleotides long. The pri-miRNAs structure consists of a short stem with a two-nucleotide 3′ overhang recognized by Exportin-5-Ran-GTP, thus inducing nuclear export of this molecule into the cytoplasm. Pre-miRNAs are then cleaved in the cytoplasm by RNase III Dicer and TRBP (double-stranded RNA-binding protein) to form miRNA duplexes. Dicer is specifically exploited in humans to help process pre-miRNAs and build the RISC. The loaded strand’s 5′ end, which includes the miRNA seed sequence, subsequently binds to miRNA recognition sites in the target gene’s 3′ translated regions. The Ago2 proteins form a complex with the functional strand of the mature miRNA to form the RISC, which causes silencing effects by translational suppression, mRNA cleavage, and deadenylation. Meanwhile, the passenger strand will be degraded. pi-miRNAs, primary transcripts; pre-miRNA, precursor miRNA; DGCR8, Drosha and DiGeorge syndrome critical region gene 8; Ran, Ras-related nuclear protein; GTP, Guanosine-5′-triphosphate; RISC, RNA-induced silencing complex; Ago2, Argonaute; MM, multiple myeloma.

miRNAs are short, non-coding RNA transcripts, and typically on average, they are 22 nucleotides in length. After transcription, the DNA sequences become pre- and mature miRNAs. miRNA interacts with target mRNA 3′ UTRs [30], 5′ UTRs, coding sequences, and gene promoters [31], causing changes in gene expression. This ncRNA is transported across subcellular compartments, specifically the cytoplasm and the nucleus, to regulate translation and transcription rates [30]. The dysregulation of miRNAs is linked to several complex diseases, such as malignant hematopoiesis, including leukemia, lymphomas, myeloma [32], and epithelial malignancies, including lung cancers and breast carcinomas [33]. Furthermore, these molecules mediate intercellular communication via cell–cell signaling [34,35]. miRNA biogenesis occurs within the nucleus of the cell (see Figure 1). First, RNA polymerase II (Pol II) initiates the primary miRNA (pri-miRNA) hairpin transcription. Drosha, an RNA polymerase III family member, then cleaves the pri-mRNA hairpin to form a 70–100 bp precursor miRNA (pre-miRNA). Subsequently, Exportin 5 transports the pre-miRNA fragment to the cytoplasm, where Dicer (another member of the RNA polymerase III family) cleaves the molecule into a 20–22 bp miRNA/miRNA duplex. The RNA helicase separates the miRNA duplex, and the mature miRNA binds to the Argsonoute protein (Ago2), forming a ribonucleoprotein (RNP) complex. The resulting miRNA-RNP complex (miRgonaute) allows the mature miRNA strand to attach to the 3′-UTR of the target mRNA. Complexes that enhance deadenylation ultimately slow the translation process [35].

### 2.2. Long Non-Coding RNAs (lncRNAs)

**Figure 2:**
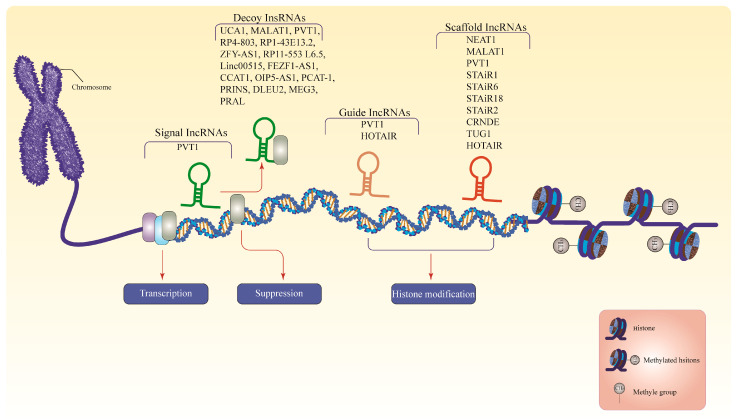
The lncRNAs modes of action. Signals from lncRNAs are dynamically translated and expressed in response to developmental inputs, regulating the simultaneous activities of transcription factors to govern gene expression. Decoy lncRNAs interact as endogenous competitive RNA molecules that remove transcription factors or other associated proteins from chromatin to inhibit the transcription of target mRNAs. The guide lncRNAs direct RNP complexes to target genes, resulting in chromatin modification. Scaffold lncRNAs assemble a cluster of enzymes to form a chromatin-modifying complex that either inhibits or activates transcription. LncRNAs, long non-coding RNAs; PVT1, plasmacytoma variant translocation 1; UCA1, urothelial cancer associated 1; MALAT1, metastasis-associated lung adenocarcinoma; ZFY-AS1, ZFY Antisense RNA 1; linc00515, long intergenic non-protein coding RNA 515; FEZF1-AS1, FEZF1 Antisense RNA 1; CCAT1, colon cancer associated transcript 1; OIP5-AS1, Opa interacting protein 5-antisense RNA 1; PCAT-1, Prostate Cancer-Associated Transcript 1; PRINS, psoriasis susceptibility-related RNA gene induced by stress; DLEU2, deletion in leukemia 2; MEG3, maternally expressed gene 3; PRAL, P53 Regulation Associated LncRNA; HOTAIR, HOX antisense intergenic RNA; NEAT1, nuclear paraspeckle assembly transcript 1; STAIR18, STAT3-induced lncRNAs 18; STAiR1, STAT3-induced lncRNAs 1; STAiR6, STAT3-induced lncRNAs 6; STAiR2, STAT3-induced lncRNAs 2; CRNDE, colorectal neoplasia differentially expressed; TUG1, taurine upregulated 1. Note: For more information, please see the text.

The lncRNAs represent the largest portion of the mammalian non-coding transcriptome [36]. The human GENCODE revealed more than 16,000 lncRNAs in the human genome [37] and is estimated to exceed 100,000 with more recent discoveries [37,38]. The lncRNAs are RNA molecules that are >200 nucleotides in length, which are not translated into functional proteins [39]. These molecules are aberrantly expressed in several cancers [36], such as Prostate Cancer-Associated Transcript 1 (PCAT-1) in prostate cancer [40], colorectal neoplasia differentially expressed (CRNDE) in colorectal cancer [41], and metastasis-associated lung adenocarcinoma (MALAT1) [42], HOX antisense intergenic RNA (HOTAIR) [43], and breast cancer anti-estrogen resistance 4 (BCAR4) [44] in breast cancer. The lncRNAs residing within the nucleus primarily involve epigenetic and transcriptional regulation, whereas their cytoplasmic counterparts often engage in post-transcriptional regulation [45]. Most lncRNAs are discovered inside the nucleus, interacting with various nuclear constituents such as the nucleoplasm, sub-nuclear domains, and chromatin, impacting the nuclear structure and function [46]. Nonetheless, several lncRNAs exist in the nucleus and cytoplasm [47,48]. The lncRNAs have four modes of function: signal, decoy, guide, and scaffold. Figure 2 summarizes the mode of action of all lncRNAs discussed in this review. Studies have revealed that lncRNAs work alone or with transcription factors to enhance gene transcription.

Generally, lncRNAs are decoy molecules that connect to miRNAs to compete with mRNA and prevent miRNA from inhibiting mRNA molecules. In this framework, lncRNAs possess miRNA response elements (MREs), which are complementary sequences to miRNAs. The MREs facilitate the binding of lncRNAs, enabling these molecules to sequester miRNAs from the intended mRNA targets. Therefore, lncRNAs prevent the binding of miRNAs to target mRNAs, thereby inhibiting translation or promoting degradation that augments the stability and expression levels of the mRNA targets. The lncRNAs can also function as decoys for proteins or protein complexes. The lncRNAs sequence contains binding sites that can interact with the target proteins. The interaction between lncRNA and proteins can potentially separate proteins from the typical binding sites in the genome, disrupting protein–protein interactions or impeding the protein’s binding capacity to specific DNA or RNA sequences. Therefore, lncRNAs could impact cellular processes, including transcriptional control, chromatin remodeling, and signaling pathways [48,49,50,51].

## 3. Dysregulation of miRNA in MM

Studies have found aberrant miRNA expression in MM, suggesting new biomarker candidates for MM therapy. MiRNAs are significantly involved in MM pathogenesis as these molecules interfere with the MM bone marrow environment, influencing target genes and altering signaling molecules and pathways. Some miRNAs function as oncomiRs, exerting a pro-cancer effect by downregulating tumor suppressor genes. Overexpression of oncomiRs in MM cells can impair gene function, promoting unrestricted cell proliferation in the bone marrow.

### 3.1. miRNA Dysregulation in the Bone Marrow Microenvironment

miR-202 has been extensively investigated and associated with MM pathogenesis, potentially influencing cellular proliferation and adhesion mechanisms. Interestingly, in certain cancers, miRNA-202 has been reported as a tumor suppressor, while in others, it may have oncogenic properties [52]. In MM, miR-220 affects MM cell proliferation and adhesion by deregulating B cell-activating factors in bone marrow stromal cells (BMSCs) [53]. Moreover, miR-202 mimics disrupt the signaling between BMSCs and MM cells by reducing the B-cell activating factor (BAFF) expression. Plasma cells rely heavily on BMSCs’ support to ensure the survival of MM cells. In MM-related BMSCs, miR-202 is substantially downregulated. MiR-202 mimics are utilized in restoring miR-202 levels, thus reducing growth-promoting activity by downregulating B-cell lymphoma 2 (BCL-2) and BAFF expressions [53]. In addition, miR-202 expression was upregulated in the blood of MM patients compared to the control group (*p* < 0.01). This finding demonstrated the significant correlation between the concentrations of serum Beta-2 microglobulin (β2M) and κ light chain and miR-202 expression (*p* = 0.0305; r = 0.358, P = 0.0348). The circulating miR-202 level is possibly an indirect indicator of tumor presence, but further research is necessary to confirm the association of these miRNAs with the changes that occur in MM. Nevertheless, there was no correlation between miR-202 expression with the lactate dehydrogenase (LDH) and λ light chain concentration. Therefore, evaluating the relative expression levels of serum miR-202 in the supplementary diagnosis of MM might be useful in a clinical setting [54].

MiR-21 is also aberrantly upregulated in MM cells attached to BMSCs, promoting cell survival and enhancing clonogenic development in stromal-free circumstances [55]. Furthermore, miR-21 significantly affects the development and progression of MM by explicitly targeting and suppressing the expression of various tumor suppressor genes, such as phosphatase and tensin homolog (PTEN), responsible for regulating cellular growth and viability. The downregulation of PTEN by miR-21 potentially results in the uncontrolled proliferation of MM cells. Moreover, using miR-21 inhibitors in MM treatment upregulated PTEN and downregulated p-Ak strain transforming (AKT) in the retrieved xenografts [55]. Suppression of miR-21 in U266 MM cells inhibited cellular proliferation and cell cycle arrest and facilitated cellular death. The protein inhibitor of the activated signal transducer and activator of transcription 3 (PIAS3) was also simultaneously inhibited, as this molecule has been identified as the specific target for miR-21. The identification of PIAS3 within the miR-21-signal transducer and activator of transcription 3 (STAT3) positive regulatory loop contributes to the understanding of the molecular underpinnings of miR-21’s biological impacts on MM cells, paving the way for the advancement of innovative treatment approaches aimed at combating MM [56].

The MM-BMSCs also demonstrated significant overexpression of miR-223, miR-485-5p, miR-519d, and miR-16. miR-223-3p has been linked to disease progression and may serve as a potential prognostic indicator in MM. Studies have demonstrated that miR-223-3p downregulation is associated with significantly shorter overall survival (OS) phases. However, more randomized trials are necessary to assess the prognostic significance of miR-223-3p in large MM cohorts [57]. A recent study has identified the significant regulatory role of miR-223 in the hematopoietic system involving hematopoietic stem cells, erythroid cells, and granulocyte–monocyte progenitors at different development stages [58]. Patients with MM who undergo autologous hematopoietic stem cell transplantation and exhibit elevated levels of miR-223-3p in their serum will likely achieve a complete response and experience longer OS [59]. Furthermore, there is a positive correlation between the expression of miR-223-3p in the blood serum at day +7 after allogeneic hematopoietic stem cell transplantation (AHSCT) and during engraftment [59]. A study reported decreased co-expression levels of miR-223 with miRNAs let-7a, miR-15a, miR-20a, miR-21, miR-106b, and miR-361 in the MM bone marrow microenvironment, peripheral blood, and CD138+ plasmocytes [60]. MiR-223 downregulation is expected as the disease progresses from monoclonal gammopathy of undetermined significance (MGUS) to symptomatic MM. Additionally, the downregulation of miR-223-3p has been observed in bortezomib-resistant MM cell lines and patients [59]. MiR-223 regulates the suppression of circ-CCT3 and inhibits bromodomain containing 4 (BRD4), thereby enhancing the responsiveness of bortezomib-resistant MM cells to bortezomib [61].

The specific role of miR-485-5p in MM is still under investigation, but the miRNA is expected to be involved in cellular survival, proliferation, and drug resistance mechanisms. The inhibition of miR-485-5p in transfected MM-BMMSCs, along with the associated cluster delta-like homolog 1 gene and the type III iodothyronine deiodinase gene (DLK1-DIO3), resulted in a decrease in cell cycle progression during the S phase and an accumulation of cells in the G1/G0 phase. The expression of miR-485-5p was downregulated when the interaction between MM-BMMSCs and MM cells was hindered, indicating that miR-485-5p may be a promising candidate for regulating senescence in MM-BMMSCs [62].

The miR-519d has been identified in regulating cell cycle progression and proliferation. MiR-519d together with miR-485-5p are in two imprinted clusters on chromosomes 14 (DLK1-DIO3) and 19 (C19MC), respectively. The overexpression of miR-485-5p and miR-519d in MM-BMMSCs is predicted to promote cell senescence. Furthermore, cell–cell interactions exhibit significantly reduced miR-519d expression by 2.72- to 2.85-fold in co-cultured KMS12-PE myeloma cells (*p* < 0.02) [62]. The expression miR-16 and miR-15a are reduced in MM cells and are associated with the MM progression to an advanced stage by inhibiting angiogenesis. Additionally, vascular endothelial growth factor A (VEGF-A) is repressed post-transcriptionally by miR-15a, imposing an antiangiogenic effect primarily through inhibiting VEGF-A expression. The presence of miR-16 and miR-15a also inhibits the tumor development and the formation of new blood vessels [63].

MiR-29b has been identified as an oncogene in multiple cancers, including MM. In the MM bone marrow microenvironment, miR-29b inhibits osteoclast differentiation and suppresses osteoclast activation [64]. The expression of miR-29b was downregulated in MM cell lines and tissues. In addition, miR-29b expression was significantly reduced in MM patients at stage III compared to patients at stage I and stage II, suggesting a potential association between miR-29b and the ISS stages of MM patients. Moreover, miR-29b inhibits MM cell proliferation by promoting cell cycle arrest and inducing apoptosis in H929 and U266 cell lines transfected with miR-29b mimics, which specifically target forkhead box P1 (FOXP1). Therefore, restoring FOXP1 expression suppresses miR-29b and leads to anti-proliferative and pro-apoptotic effects in MM cell lines. Furthermore, the inhibitory action of miR-29b on the growth of MM tumors has been demonstrated in a mice model [65]. Elevated miR-29b in dendritic cells (DCs) suppressed the activation of pro-inflammatory pathways mediated by STAT3 and Nuclear factor kappa B (NF-κB) and cytokine/chemokine signaling networks when co-cultured with MM cells. This correlation is associated with poor patient outcomes and supports the occurrence of bone disease. In addition, miR-29b inhibits interleukin (IL)-23 in vitro and in the SCID-synth-hu in vivo model. MiR-29b also acts as an antagonist to counteract T-helper 17 (Th17)-mediated inflammatory responses. In a different study, miR-29b demonstrates significant anti-proliferative effects and disrupts the genomic stability of MM cells. This process hindered the DCs’ ability to support the growth and survival of MM cells and altered their functional phenotype, which is linked to the inhibition of miR-29b. Therefore, miR-29b is a promising candidate for miRNA-based immunotherapy for MM [66].

The MM cells rely on the intricate interplay with the bone marrow microenvironment to sustain their proliferation and longevity. The tumor suppressor miRNA, miR-138, potentially regulates these interactions by selectively targeting adhesion molecules and factors pivotal in facilitating communication between MM cells and BMSCs. Resultantly, this process influences the adhesion and migration of MM cells within the bone marrow. In contrast, inhibiting miR-138, which was previously elevated in MSCs and MM cells, improved bone growth in the bone marrow niche [67]. Moreover, another study reported that miR-138-5p plays a role in the osteogenic differentiation of MM-MSCs by targeting and reducing three specific genes, namely Rho Associated Coiled-Coil Containing Protein Kinase 2 (ROCK2), Transcriptional Repressor GATA Binding 1 (TRPS1), and Sulfatase (SULF2), which are associated with osteochondrogenesis [68].

Exosomes are responsible for transporting and secreting essential miRNA to intercept the regulatory systems in a tumor microenvironment. The miRNA expression profile in exosomes secreted by BMSCs derived from MM bone marrow significantly differed from healthy individuals. For example, Manier et al. have identified two types of circulating exosomal miRNAs, let-7b and miR-18a, which were significantly associated with progression-free survival (PFS) and OS in a univariate study. These associations remained statistically significant even after correcting confounding factors, such as ISS and unfavorable cytogenetics in the multivariate analysis. Circulating exosomal miRNAs may be useful for detecting newly diagnosed MM patients at a higher risk for unfavorable prognoses [69].

The miR-214-3p and miR-27b-3p expressions were detected at the commencement of MM progression, which is associated with reduced apoptosis and resistance to the apoptosis mechanism, thus enhancing the proliferation of myeloma fibroblasts. In contrast, myeloid cell leukemia-1 (MCL1), an antiapoptotic factor, decreases when miR214-3p and miR-27b-3p are inhibited and induce cellular apoptosis [70]. Upregulation of miR-146a was observed in human MSCs after exposure to the conditioned medium derived from MM cells. Subsequently, cytokine and chemokine expressions were upregulated, specifically IL6, IL-8 secretion and CXC Motif Chemokine Ligand 1 (CXCL1), Interferon gamma-induced protein 10 (IP-10), monocyte chemoattractant protein-1 (MCP-1), and C-C Motif Chemokine Ligand 5 (CCL-5) expression. This event augmented the viability and migratory capacity of MM cells [71].

Upregulation of miR-152 expression was reported in the bone marrow microenvironment, which was associated with downregulation of Dickkopf-1 (DKK1) proteins of MM.1S cells that were exposed to miR-152. The miR-152 expression also increased in MM1.S mice treated with miR-152, which was associated with elevated bone mineralization through the activation of DKK1 in MM and ultimately enhanced bone formation [72].

### 3.2. miRNAs Dysregulation Causes Genomic Instability

A prospective targeted therapy for the treatment of MM could involve miRNAs due to the ability to dysregulate genomic stability. Qin et al. [73] reported that miR-137 targeting Aurora kinase A (AURKA) demonstrated a favorable epigenetic connection between miR-137 (tumor-suppressive miRNA) and PFS in MM patients. The diminished expression of miR-137 in MM is caused by significant DNA methylation events occurring in the promoter region of miR-137, which consequently hinders its transcription and leads to reduced expression. In MM patients, hypermethylated miR-137 indicates a higher risk of IgH translocations (4;14). The expression of aberrant miR-137 decreases the frequency of deletions at chromosomal 1q21 gains and 1p22.2, 14q, and 17p13. Additionally, the upregulation of miR-137 enhances the sensitivity towards bortezomib and epirubicin in a laboratory setting. Furthermore, miR-137 tends to be epigenetically repressed in MM [74]. Interestingly, miR-137 possibly mitigates drug resistance and addresses the chromosomal instability of MM cells when functionally restored by modulating the apoptosis and DNA damage pathways [73].

The miR-150 and miR-22 overexpression inhibits alternative nonhomologous end-joining (ALT-NHEJ), repairing DNA double-strand breaks and increasing genomic instability [75]. Bong et al. observed that miR-150 regulates two cell cycle-associated genes, RAD54 Like (RAD54L) and Cyclin A2 (CCNA2) [76]. RAD54L is responsible for repairing the DNA double-strand breaks and modifying chromatin during the G1/S transition through homologous recombination [77]. When the DNA is wrongly repaired, this event can lead to various genetic alterations, including mutations, deletions, and oncogenic translocations in human cells [77,78]. In MM, RAD54L is overexpressed, an abnormality that might be attributed to miR-150 downregulation. Meanwhile, CCNA2 is a known regulator of cell cycle pathways, serving as a genetic marker for predicting prognosis and outcomes in MM and other types of cancer [76].

miR-22 demonstrates tumor-suppressing activity in vitro and in vivo by targeting DNA ligase III (LIG3) in MM. The ectopic expression of miR-22 significantly inhibited LIG3-mediated nuclear and mitochondrial DNA (mtDNA) repair, significantly increasing the unrepaired DNA damage and, ultimately, the apoptosis of MM cells. Furthermore, the ectopic expression of miR-22 significantly inhibits the Alt-NHEJ repair mechanism, significantly reducing new genetic alterations in MM cells. This outcome suggests that miR-22 exerts a protective role against genomic instability, promoting the disease progression and development of drug resistance [79].

### 3.3. miRNAs Dysregulation Impacts the Immune Response

miRNAs are also a significant modulator of immune response in MM via numerous immune cells and pathways. For instance, miR-125b exhibits anti-MM and tumor-suppressor activity in vitro and in vivo by significantly downregulating interferon regulatory factor 4 (IRF4) expression. The IRF4 is essential in the maturation and differentiation of regulatory T cells, Th2, Th9, and Th17 cells [80]. When IRF4 is suppressed, the activities of c-Myc, caspase-10, and cellular FLICE-like inhibitory protein (cFlip) are subsequently downregulated. These proteins are key IRF4-downstream effectors that improve anti-tumor activities and enhance survival rates in MM. In addition, miR-29b demonstrates remarkable anti-MM activity by promoting Interferon (IFN) to regulate the differentiation of Th1 cells [81]. Another study found that inhibiting miR-21 in IT cells decreases Th17 differentiation, revoking ’Th17’s ability to promote MM cell proliferation and osteoclast activity [82].

miRNAs could also be implicated in the IL regulation. For instance, the oncogenic effects of IL-17 suppress miR-192 in MM cells. Moreover, IL-17 activates the oncogenic p65 transcription factor, resulting in miR-192 inhibition by suppressing the miR-192 promoter. Thus, miR-192 and IL-17 or IL-17RA expressions are inversely correlated in MM patients’ bone marrow [83]. An earlier study revealed that IL-17, IL-21, and IL-27 were upregulated, and IL-22 was downregulated in the peripheral blood of MM patients compared to the healthy controls. Therefore, the bone marrow mononuclear cells of MM patients are linked to higher miR-181a and lower miR-15a/16, miR-34a, and miR-194 expressions [84].

The scope of miRNA regulation has been expanded to the regulation of natural killer (NK) cell-induced antitumor activity through the modulation of stress-induced major histocompatibility complex class I polypeptide-related sequence A and B (MICA & MICB), UL16-binding proteins (ULBPs), cytotoxic enzyme, and receptors [85]. The overexpression of miR-10b in MM cells inhibited MICB expression, lowering the chances of cell-mediated lysis. This outcome was achieved by targeting tumor cells in laboratory settings and living organisms using NKG2D or KLRK1 (Killer Cell Lectin-Like Receptor K1), an activating receptor. The NKG2D is a transmembrane protein belonging to the NKG2 family of C-type lectin-like receptors that acts as a regulatory mechanism for activating NK cells [86].

A regulatory interplay between miRNAs and DCs has received much attention within the context of MM. The role of DC in bone marrow infiltrations of MM patients perpetuates the MM cell proliferation and hijacks the apoptotic process following treatment with melphalan and bortezomib [87]. The DCs are characterized by miR-301a overexpression, which induces IL-12 and IL-6 and Tumor necrosis factor (TNF) secretion to stimulate the T-cell responses [88]. In addition, miR-155 and miR-221 stimulate IL-12 activity in DCs, activating the release of pro-inflammatory cytokines such as IL-6 and TNF [89]. miR-22 exerts an inhibitory effect on p38 protein expression by directly binding to the 3’ UTR region of the p38 mRNA, disrupting IL-6 synthesis and Th17 cell differentiation driven by DCs. The miR-22 upregulation in DCs may reduce the suppression of tumor growth. Conversely, miR-22 downregulation reverses this impact and enhances the therapeutic efficacy of DC-based immunotherapy [90]. The overexpression of miR-21 and miR-155 in MM generates more myeloid-derived suppressor cells (MDSCs) as a response to the targeting of SH-2 containing inositol 5′ polyphosphatase 1 (SHIP-1) and PTEN, respectively [91]. This occurrence is prevalent in the peripheral blood and the patient’s bone marrow responsible for osteoclast-like cell development in MM-associated lytic bone presentation [24]. In addition, osteoclast-like cell development in MM-associated lytic bone presentation can suppress the MDSCs activity, diminishing and limiting the CD4 and CD8 T-cell responses [92].

Macrophages are critical components of the MM cell biology. In the relapse and refractory phases of active MM, inflammatory factors such as vascular endothelial growth factor (VEGF), fibroblast growth factor 2 (FGF-2), and hepatocyte growth factor (HGF) are activated and recruited, leading to macrophage activation. In the bone marrow, macrophages shielded MM cells from spontaneous apoptosis elicited by melphalan therapy [80]. miR-155 directly targets the suppressor of cytokine signaling 1 (SOCS1) [93], BCL6 transcription repressor (BCL6) [94], and interleukin 13 receptor subunit alpha 1 (IL13RA) [95], promoting macrophage activation to trigger the pro-inflammatory response. Meanwhile, miR125b stimulates macrophages, substantially downregulating macrophages in response to inflammatory stimuli [96]. Furthermore, miR-187 activates macrophages in an IL10-dependent mode, and an increase in this miRNA expression inhibits LPS-induced TNF-, IL-6, and IL12p40 transcription, indicating the importance of miR-187 in suppressing the inflammatory response [97].

### 3.4. miRNAs Dysregulation in Therapy-Resistant

miRNAs potentially enhance MM cells sensitivity to bortezomib, melphalan, and dexamethasone treatments. For example, miR-29b causes MM cells to become resistant to bortezomib by activating the feedback loop of the SP1 transcription factor [81]. The co-treatment of bortezomib and miR-29b induces significant anti-MM and pro-apoptotic effects in synthetic miR-29b transient or the stable lentivirus-enforced expression due to the phosphoinositide 3-kinase/protein kinase B (PI3K/AKT) pathway. Moreover, MM xenografts consistently expressing miR-29b reduce the tumorigenic ability, indicating the regulatory role of miR-29b in the loop and potential pharmacological interventions in the future [81].

Zhang et al. [98] provided insights into the expression patterns of exosome-associated miRNAs, which can be utilized as predictive markers for bortezomib resistance in MM patients. This study discovered 3180 MM-related miRNAs, where 83 were upregulated and 88 were downregulated. The expression levels of miR-513a-5p, miR-20b-3p, and let-7d-3p were significantly upregulated, while miR-125b-5p, miR-19a-3p, miR-21-5p, miR-20a-5p, miR-17-5p, miR-15a-5p, and miR-16-5p were downregulated. Notably, most miRNAs (>90%) exhibit novel functions that warrant further investigations. The differential expression patterns of miRNAs suggest the regulatory roles in post-translational pathways and other transcription factors, such as the MAP kinase pathway and ubiquitin-conjugating enzyme activity. The gene ontology indicates a synergistic network between miRNA and RNA, revealing the post-transcriptional regulatory network responsible for bortezomib resistance in MM patients. This network is primarily governed by miR-17-5p, miR-20a-5p, miR-15a-5p, and miR-16-5p. miR-15a and miR-16 are located at 13q13.4, while miR-17-92 is found on chromosome 13q31.3, and all correlated with unfavorable prognosis in approximately 50% of MM patients. Nevertheless, the gene expression remains unaffected by the loss of chromosome 13. Therefore, additional prospective studies on a bigger cohort are required to ascertain the expression of exosome-associated miRNA in MM-associated bortezomib-resistant patients [98].

miR-27a was significantly reduced in bortezomib-resistant MM cells compared to the normal MM cells. Bortezomib sensitivity can be restored through the ectopic expression of miR-27a, which effectively inhibits the expression of the cyclin-dependent kinase 5 (CDK5) oncogene [99]. A different study revealed that the endogenous levels of CDK5 mRNA were significantly decreased within MM cells that were transfected with miR-27a-5p. However, no corresponding alteration was observed in the levels of CDK5 protein [99].

Elevated miR-137 and miR-197 levels in MM cell lines and patients’ cells act as oncomiRs by increasing MM cell viability while decreasing its sensitivity to bortezomib [74]. Gutiérrez et al. observed that the expression of miR-137/197 was significantly reduced in a group of 60 MM patients than plasma cells from healthy donors [100]. Furthermore, the expression of the myeloid leukemia 1 (MCL-1) gene is dysregulated in intact MM cells. An increase in MCL-1 expression has been linked to relapse and poor survival [101], besides intercepting various proapoptotic signals to significantly promote MM cell survival [102]. Meanwhile, Yang et al. reported that activated miR-137 and miR-197 initiate apoptosis and inhibit tumorigenicity via direct binding to the 3′ UTR of MCL-1 in MM cell lines. The MCL-1 is significantly downregulated in MM cell lines, resulting in significant antiproliferative and proapoptotic effects. For example, the miR-137/197 complex promoted BH3 proapoptotic proteins (BAD, BAX, and BID) while simultaneously suppressing antiapoptotic proteins (BCL-2, BCL-XL, and surviving). Additionally, the downregulation of MCL-1 is also evident in couples with a decrease in colony formation and cellular motility [74].

Overexpression of miR-202 alters the Jun amino-terminal kinases/Stress-activated protein kinases (JNK/SAPK) signaling pathway, improving the sensitivity towards bortezomib while decreasing the sensitivity to dexamethasone and thalidomide [103]. In cellular models resistant to MM-dexamethasone, suppressing miR-221/222 expression prevents dexamethasone resistance and activates the ATG12/P27-mTOR pathway, which regulates autophagy [104]. It was also revealed that the levels of miR-193a expression serve as a direct target for lncRNA nuclear paraspeckle assembly transcript 1 (NEAT1), resulting in an inverse association with the dexamethasone sensitivity of MM cells [105]. miR-125 exerts an impact on the p53 tumor suppressor and the expression of the anti-apoptotic protein SIRT1, which potentially contributes to the development of resistance in MM cells against cytotoxic effects induced by dexamethasone [106]. Furthermore, MM cells can intercept the apoptotic pathway induced by dexamethasone due to miR-125b expression, thus reducing their susceptibility to the drug [106].

On the other hand, miR-137 decreases the expression of protooncogene-encoded c-Met protein (c-MET) and prevents Akt phosphorylation, enhancing MM cells’ sensitivity to dexamethasone. The expression of miR-137 is reduced in MM cell lines and in the CD138+ bone marrow mononuclear cells of MM patients by targeting the melanocyte-inducing transcription factor (MITF). However, when miR-137 expression was elevated, it did not exert a significant effect on the expression of serine/threonine protein kinase (AKT). However, there was a significant decrease in the expression of MITF, c-MET, p-AKT, and its phosphorylated substrate protein, which is associated with p53 upregulation. Furthermore, the overexpression of miR-137 or MITF-shRNA leads to a significant enhancement in the sensitivity of MM cells to dexamethasone [107].

A positive correlation between miR-221/222 upregulation and melphalan resistance in MM cells has been reported. In contrast, inhibiting miR-221/222 enhanced melphalan sensitivity and apoptosis in MM cells. This outcome was evident in nonobese diabetic/severe combined immunodeficiency (SCID/NOD) mice implanted with human melphalan-resistant MM xenografts and treated with systemic LNA-i-miR-221 and melphalan. Various biological factors contribute to this outcome, including the upregulation of the proapoptotic Bcl-2-binding component 3/p53 upregulated modulator of apoptosis (BBC3/PUMA), the alteration of drug influx–efflux transporters solute carrier family 7 member 5 (SLC7A5/LAT1), and the presence of the ATP-binding cassette transporter multidrug resistance-associated protein 1 (ABCC1/MRP1). In summary, miR-221/222 is a promising drug-sensitizing agent [108].

The downregulation of miR140-5p has been linked to an inferior autophagy mechanism [109]. The regulatory influence of miR-140-5p on MM cell lines, U266 and RPMI 8226, was observed through the targeting of vascular endothelial growth factor A (VEGFA). The transfection of miR-140-5p mimic in MM cell lines demonstrates a significant reduction in cell survival, migration, and invasion, ultimately promoting cell apoptosis. This impact is achieved through a notable drop in the expression levels of Ki-67, cyclin D1, vimentin, Snail, matrix metalloproteinase (MMP)-2, and MMP-3 [110].

Meanwhile, the atypical dysregulation of miR-203 is associated with the overexpression of exogenous RecQ-like helicase 1 (RECQ1), which is responsible for DNA repair and genomic stability maintenance. The RECQ1 exerts a protective effect on MM cells by shielding them from the bortezomib- and melphalan-induced cytotoxicity [111]. Du et al. reported that MM side population (SP) cells exhibit a unique miRNA expression pattern. The study also highlighted the significant roles of miR-451, miR-144, and miR-150 in the pathological mechanism of cancer stem cells (CSCs). The expression of miR-451 in a constitutive manner interferes with the phosphoinositide 3-kinase/protein kinase B/mammalian target of rapamycin (PI3K/Akt/mTOR) signaling pathway by targeting the 3′UTR of tuberous sclerosis 1 (TSC1). Consequently, TSC1 is downregulated, leading to the suppression of ribosomal Protein S6 (S6) and eukaryotic translation initiation factor 4E (eIF4E)-binding protein 1 (4EBP1) protein phosphorylation. miR-451 inhibitor impaired the expression of multidrug resistance 1 (MDR1) mRNA in MM SP cells, potentially contributing to the synergistic effect between the miR-451 inhibitor and MDR1 mRNA. The efficacy of anti-myeloma agents can be modulated by inhibiting the expression of specific transcription factors involved in suppressing the MDR1 gene activation [112].

miR-152 enhanced the sensitivity of MM cells to melphalan, increasing the rate of cellular apoptosis when the miR-152 mimic was introduced [72]. The co-existence of miR-152 mimic and melphalan significantly promoted apoptosis in MM.1S and OP-M2 cells, suggesting a synergistic pro-apoptotic action through the induction of poly(ADP-ribose) polymerase (PARP) cleavage [72].

### 3.5. miRNAs Dysregulation in Disease Prognosis

**Figure 3:**
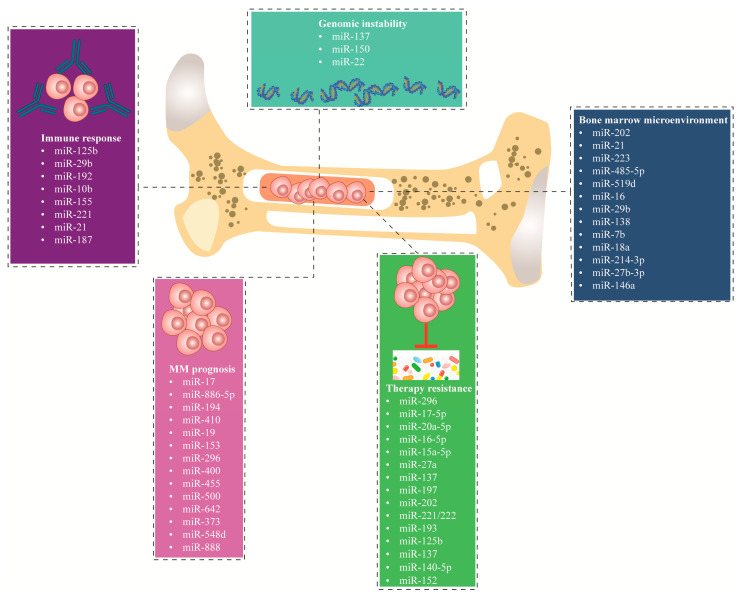
miRNAs demonstrated as significant key players in dysregulating multiple molecular mechanisms in MM, including dysregulation of the bone marrow environment, genomic instability, immune response, MM prognosis, and therapy resistance. MM; multiple myeloma.

miRNAs significantly affect the prognosis of MM patients. Wu et al. [113] reported a significant association between elevated levels of miR-17 and miR-886-5p and decreased OS in MM patients. The miRNAs that exhibited the highest discriminative power for OS were also identified as outcome classifiers, thereby dividing the patient cohort (n = 163) into three distinct risk groups. The first group, classified as high risk, consists of individuals with elevated expression levels in both categories, accounting for 13.5% of the total patients. Next, the middle risk includes patients with high expression levels in either one of the categories, representing 54% of the cases. Lastly, the third group, identified as low risk, comprises individuals with low expression levels in both categories, making up 32.5% of the cases. Furthermore, the mir-17 and miR-886-5p molecules were shown to be strongly correlated with the enhanced predictive capability of the in-situ sequencing/fluorescence in Situ Hybridization (ISS/FISH) method (P = 0.0004). This correlation remained statistically significant even after accounting for prognostic markers generated from gene expression profiling (P < 0.002) [113]. Furthermore, there is a significant association between the downregulation of miR-15a, disease progression, and patient survival. The low expression of miR-15a in newly diagnosed MM patients is a critical prognostic factor. Patients with low miR-15a expression (<2.35) had significantly shorter PFS and OS compared to patients with high miR-15a expression (≥2.35). Therefore, in multivariate analyses, miR-15a continues to be identified as an independent prognostic factor for short PFS and OS in newly diagnosed MM patients [114]. A separate study exhibited an augmentation in the expression of miR-410 in both newly diagnosed MM patients and in relapsing tissues and cell lines. Interestingly, the clinical investigation demonstrated a positive correlation between miR-410 and the advanced ISS stage. Furthermore, it was observed that MM patients with elevated levels of miR-410 had significantly reduced PFS and OS [115]. Hao et al. demonstrated the association between miR-19a expression and poor prognosis MM was not influenced by genetic abnormalities. The study indicates a significant correlation between the downregulation of miR-19a and the advancement of the international staging system. This correlation is notably observed in cases with del(13q14) and 1q21 amplification. Furthermore, the downregulation of miR-19a is associated with a reduction in both PFS and OS rates. Nevertheless, patients exhibiting decreased levels of miR-19a demonstrated enhanced responsiveness to bortezomib treatment and exhibited a notable increase in OS following therapy with bortezomib. Hence, miR-19 serves as a significant prognostic indicator for the identification of high-risk MM cases [116]. In a different study, the downregulation of miR-153, miR-296, miR-490, miR-455, miR-500, and miR-642 and high expression of miR-548d, miR-373, miR-554 and miR-888 were associated (P < 0.05) with event-free survival in MM patients. On the contrary, the overexpression of miR-373, miR-548d, miR-554, and miR-888 was predicted to result in poor patient prognosis [117]. Figure 3 summarizes the miRNAs discussed in this review according to their molecular mechanism in MM. Table 1 summarizes the expression level and functional role of distinct miRNAs associated with MM.

## 4. Dysregulation of lncRNAs in MM

In recent years, numerous studies have focused on the role of lncRNAs in MM pathogenesis. Since then, the role of aberrantly expressed lncRNAs in MM has been reported, highlighting the potential in clinical applications. For instance, lncRNAs participate in the differentiation of erythroid, myeloid, and lymphoid cells and the regulation of blood cell growth and longevity, hence supporting the role of this molecule in hematological disorders development [119]. The lncRNAs are regulators of gene expression mRNA translation and are involved in various routes and mechanisms such as epigenetic regulation of gene expression, governing interaction with RNA-binding proteins, and regulating miRNA. Furthermore, lncRNAs are tumor-related genes that can function as oncogenes or tumor suppressors, thus promising biomarkers for novel therapeutic drugs [120].

Xu et al. [121] suggested that CEACAM1 could potentially function as a tumor suppressor in MM. The observed elevation of CEACAM1 was shown to be associated with a notable decrease in the proliferation of MM cells, induction of cell death, and inhibition of cell invasion and migration. These effects were probably mediated through the activation of caspase-3 and the downregulation of matrix metalloproteinase-2 (MMP-2) and matrix metalloproteinase-9 (MMP-9). From the patient’s standpoint, CEACAM1 expression was higher in Stage I MM patients (61.5%), followed by Stage II (21.1%) and Stage III (22.2%). Furthermore, MM patients with β2-microglobulin levels equal to or less than 3.5 mg/L exhibit increased expression of CEACAM. This finding suggests that CEACAM may serve as a potential biomarker for improved prognosis in MM [121]. Meanwhile, Lu et al. [122] discovered two novel lncRNAs that act as MM oncogenes: MSTRG.155519 and MSTRG.13132 are involved in the downstream pathway of targeting carcinoembryonic antigen-related cell adhesion molecule 1 (CEACAM1) and terminal nucleotidyltransferase 5C (FAM46C), respectively. The CEACAM1, also known as CD66a, is an aberrantly overexpressed protein on the surface of plasmacytes MM. CEACAM1 exhibits a distinctive attribute wherein it assumes two distinct functionalities in diverse cancer types. Additionally, FAM46C induces growth arrest and apoptosis in MM cells and is required for disease onset, progression, and apoptosis in MM. Overexpression of FAM46C downregulates IRF-4 and MYC, leading to a decline in MM cell survival [122]. In addition, high lncRNA deletion in leukemia 2 (DLEU2) is significantly linked to miR-15a and miR-16-1 expression in MM patients with del13 [123]. At the translational level, DLUE2 displayed tumor suppressor activity by interacting with G1 cyclins E1 and D1 via miR-15a/miR-16-1, ultimately suppressing the rapid advancement of the cell cycle mechanism. Furthermore, DLEU2 inhibits cell proliferation, differentiation, and apoptosis by reducing MM DNA synthesis, cell adhesion, and cell cycle progression [124].

A significant change in the expression of the lncRNA urothelial cancer associated 1 (UCA1) was observed in MM patients, which correlates with several parameters such as blood albumin levels, monoclonal immunoglobulin, chromosomal abnormalities, and survival rates. When UCA1 is overexpressed in MM, there is a potential positive regulation of the cell cycle via cAMP Response Element-Binding Protein (CREB) regulation. The UCA1 expression is upregulated in MM patient samples and cell lines and linked to poor prognosis. Downregulation of UCA1 substantially reduced cell proliferation and increased apoptosis. Furthermore, it has been observed that UCA1 plays a significant role in the regulation of transforming growth factor-beta (TGF-β) in MM. Specifically, the overexpression of TGF-β diminished the effects of UCA1 knock-down [125].

Dysregulation of the NEAT1 lncRNAs is characterized by overexpression and linked to higher resistance to dexamethasone by targeting the miR-193a/MCL1 (myeloid cell leukemia-1) pathway [105]. Increased NEAT1 expression promotes oncogenic activity in MM and induces MM cells progression to the aggressive phase via nutrient deprivation or a hypoxic milieu. Conversely, NEAT1 suppression diminished the impact on the DNA damage repair (DDR) pathway, reducing the proliferation and lifespan of MM cells in vitro and in vivo [126].

The gradual decline in lncRNA KIAA0495 (PDAM/TP73-AS1) distinguishes normal plasma cells from benign MGUS and symptomatic myeloma [127]. The LP-1 and OCI-MY5 MM cells treated with 5-aza-2′-deoxycytidine (5-azadC) exhibited partial methylation of the KIAA0495 gene, leading to progressive demethylation of the KIAA0495 promoter [128]. Another study found a negative correlation between KIAA0495 methylation and expression patterns in MM cell lines, primary oligodendroglial tumor cells, and glioma cell lines [129]. Nonetheless, no KIAA0495 methylation was detected in primary MM patient samples at the time of diagnosis and relapse, suggesting that the methylation of tumor suppressor miRNAs or lncRNAs was not acquired in vitro. Therefore, it can be concluded that this methylation is not pathogenic. The KIAA0495 gene is found at the chromosomal location 1p36 and is frequently deleted in patients with newly diagnosed multiple myeloma (NDMM) [128]. Furthermore, KIAA0495 deletion in MM is potentially caused by haploinsufficiency and chromosome deletion [130].

The overexpression of MALAT1 lncRNA is another factor that mediates the transformation of normal plasma cells into MM. However, MALAT1 dysregulation was not significantly related to the treatment outcomes of MM patients. Downregulation of MALAT1 is related to the downregulation of genes involved in the proteasome pathway, indicating that this lncRNA may be a feasible biomarker in MM [131]. The overexpression of MALAT1 in the bone marrow microenvironment possibly provides favorable conditions for the proliferation of myeloma cells. Moreover, MALAT1 expression experiences a shift at each stage throughout the disease. The MM patients with low MALAT1 expression have an increased risk of early disease progression. MALAT1 also elevated miR-291/b-1, which is negatively linked with the mRNA expression of enhancer of zest homolog 2 (EZH2) [132].

In most human cancers, including MM, lncRNA plasmacytoma variant translocation 1 (PVT1) corresponds with c-Myc. The transcription of PVT1 is augmented by the c-Myc protein, and this lncRNAs was elevated in breast cancer patients, characterized by accelerated cellular advancement and unfavorable survival outcomes [133]. In addition, the upregulation of PVT1 and c-Myc is observed in several MM cell lines. The PVT1 rearrangements are also linked to MM with 8q24 rearrangements, indicating tumor progression. PVT1 has been coined as a target responsible for MM rearrangement, as earlier research demonstrated breakpoints within a region centromeric to PVT [134].

Other lncRNA, such as RP4-803, RP1-43E13.2, ZFY Antisense RNA 1 (ZFY-AS1), and RP11-553 L6.5, have been linked to the onset and progression of MM. These ncRNAs participate in genetic and epigenetic modifications in MM, including chromatin remodeling, DNA replication and repair, and RNA processing. Furthermore, these lncRNAs are used to classify MM patients into high-risk and low-risk categories based on OS [135]. Another study has found a negative correlation between PVT1 and miR-203a expression in MM samples, indicating a statistically significant relationship (*p* < 0.05). Additionally, it has been observed that inhibiting PVT in an in vitro setting leads to a decrease in MM cell proliferation and an increase in apoptosis (*p* < 0.05). These findings suggest that miR-203a may play a role in the regulation of PVT1 and its impact on MM progression [136].

The STAT3-dependent carcinogenesis is aided by lncRNAs in MM [135]. In INA-6 MM cells, IL-6 stimulation by STAT3 resulted in five STAT3-induced lncRNAs (STAiRs) including STAiR1, STAiR2, STAiR6, STAiR15, and STAiR18. STAiR1, 2, and 6 exhibited myeloma-specific expressions and possibly have a myeloma-specific role in sustaining MM’s integrity [137]. STRAiR2 inhibits deleted in colorectal cancer (DCC’s) activity as a tumor suppressor gene [137], whereas increased STAiR18 expression promotes chromatin silencing by engaging with epigenetic modification to the DNA packaging protein Histone H3 (H3K27me3) [138]. Likewise, STAiR18 engages with HOTAIR to switch off transcription, regulate heterochromatin, and modulate other epigenetic aberrations [138]. Meanwhile, STAiR15 expression is upregulated in the nucleus of MM cells, but more research is required to determine the function of this lncRNA [138].

The maternally expressed gene 3 (MEG3) lncRNA is often deleted in most MM patients [139]. The loss of this tumor suppressor leads to the suppression of the p53 protein, which in turn interferes with the mechanism for osteogenic differentiation of MSCs in patients with MM [140]. The MEG3, an endogenous competitive RNA, could compete with miR-181a to prevent tumor growth. In addition, the homeobox gene A11 (HOXA11), a target mRNA of miR-181a, has the potential to be favorably regulated by MEG3 through the sponging miR-181a competitively in vitro [139]. Other findings indicated that miR-140-5p inhibition causes the lncRNA coding RNA 515 (linc00515) to stimulate the expression of the protein-coding gene Autophagy Related 14 (ATG14), resulting in melphalan-resistance in MM cells. Conversely, the decrease in linc00515 inhibits ATG14 expression and reduces autophagy in MM cells [109].

The expression of the FEZF1-AS1 lncRNA is associated with a poor prognosis in MM patients. This lncRNA is an example of conflicting endogenous RNA in MM cells that disrupts the miR-610/Akt3 axis and stimulates MM cell proliferation. In contrast, FEZF1-AS1 inhibition causes cell cycle arrest at gap 0/1 (G0/G1) in MM cells, preventing cell proliferation and increasing apoptosis [141]. Meanwhile, the P53 Regulation Associated LncRNA (PRAL) lncRNA, which targets miR210, increases the sensitivity of MM cells to bortezomib, suppresses cell proliferation, and increases apoptotic rates. PRAL/mir-210 also inhibits bone morphogenetic protein 2 expression (BMP2). As a result, the PRAL/miR-210/BMP2 axis has become a crucial component in MM pathogenesis and is defined in the MM ISS and the Durie–Salmon stage in MM patients [142].

The CCAT1 also plays a role in MM progression. The oncogenic activities of these lncRNAs are characterized by the increasing expression of HOXA1 through the sequestration of miR-181a-5p. These molecular interactions are associated with lower OS rates in MM patients. Nevertheless, inhibiting CCAT1 inhibited cell proliferation, increased the apoptosis rate, and decreased tumor growth in vivo [143]. Meanwhile, Opa interacting protein 5-antisense RNA 1 (OIP5-AS1) (tumor suppressive lncRNA) binds with miR-410 to prevent its expression, thus boosting cell proliferation and cellular activities by Krüppel-like factor 10 (KLF10)/PTEN/Akt axis in MM. The OIP5-AS1 functions as a sponge or decoy for miR-410 by binding to the miRNA, impeding the interaction with the intended mRNA targets. miR-410 in the unsequestered state can target mRNAs specific to the KLF10/PTEN/AKT signaling pathway [115].

The overexpression of the oncogenic lncRNA Colorectal Neoplasia Differentially Expressed (CRNDE) in MM cells reflects the disease progression and the short survival rate of MM patients. The CRNDE functions as a competitive endogenous RNA and behaves antagonistically to the expression of miR-451 [144]. The CRISPR-mediated deletion of the CRNDE locus in MM cells results in notable reductions in proliferation and adhesion properties. Consequently, this intervention enhanced the sensitivity to dexamethasone and effectively suppressed tumor growth in an in vivo xenograft model. The findings indicate that the deletion of CRNDE in MM cells could activate various genes associated with MM development, such as IL6R. Moreover, the deletion of the CRNDE locus results in a reduction of IL6 signaling and the growth outcomes observed in MM cells [145].

Zhang et al. [146] discovered that the overexpression of lncRNA taurine upregulated 1 (TUG1) in MM patients is critical in modulating disease development. The MM patients who exhibit elevated TUG1 expression suffer from a poor prognosis [146], possibly due to the interference by tumor-suppressive miR-34a-5p that disrupts cell proliferation, apoptosis, and cell cycle pathways [147]. Additionally, Guan et al. revealed that the elevated expression of the lncRNA HOTAIR promotes MM chemoresistance to dexamethasone by targeting the Janus kinase 2/signal transducer and activator of transcription 3 (JAK2/STAT3) signaling pathway, thus increasing cell survival and the apoptotic rate [148].

Shen et al. [149] reported that the expression of the lncRNA PCAT-1 was significantly dysregulated in MM patients compared to the healthy control, with high sensitivity (71.7%) and specificity (93.8%). PCAT-1 exhibited the capacity to prevent miR-129 activity, leading to the downregulation of miR-129. Meanwhile, PCAT-1 knockdown prevented the tumor progression when miR-129 was inhibited, potentially achieved by suppressing Mitogen-activated protein kinase kinase kinase 7 (MAP3K7) expression and activating Nuclear factor-kappa B (NF-κB) [150]. In addition, PCAT-1 overexpression in MM leads to tumor cell survival by stimulating the JNK/MAPK pathways, prolonging MM cell survival [151]. PCAT-1 is essential in MM cell proliferation, rescuing the cell cycle at the S phase and inducing the apoptotic mechanism. Interestingly, the combination of PCAT-1 inhibitors and bortezomib demonstrated a robust inhibitory effect on MM cells compared with the negative control or treatment with bortezomib alone [151].

The lncRNA H19 was also positively correlated with MM and potentially linked to the disease advancement. The overexpression of lncRNA H19 has been found in the development of resistance to the drug bortezomib in MM patients via interaction with miR-29b-3p to target MCL-1 [152]. H19 is significantly upregulated in the bone marrow of MM patients, whereas lower H19 levels are observed in samples obtained from MGUS or smoldering multiple myeloma (SMM) patients. Furthermore, several MM cell lines (OPM-2, U266, KM3, XG1, JJN3, RPMI, U1996, H929, and MM1S) exhibited significantly higher H19 expression than the peripheral blood mononuclear cells (PBMCs) obtained from a pool of three healthy individuals serving as the normal reference group [153].

Aberrant overexpression of H19 was observed in both MM cell lines and sorted CD138+ MM bone marrow tissues. Suppression of H19 expression by short hairpin RNA (shRNA) in MM1S and RPMI cells results in a substantial decrease in cell proliferation and viability, as well as a reduction in colony formation in MM cells. The dysregulation also impacted the activation of NF-κB signaling by decreasing the levels of p-IκBα, nuclear P65, and the production of cytokines IL-8 and IL-6 [153].

Sedlarikova et al. [154] demonstrated a significant dysregulation (*p* < 0.05) of exosomal lncRNA psoriasis susceptibility-related RNA gene induced by stress (PRINS) in individuals with MM but not in MGUS when compared to a healthy control group. The expression of PRINS as a regulatory RNA is correlated with chromosomal aberrations commonly observed in MM, including gain (1) (q21), del (13) (q14), del (17) (p13), t (4;14), and hyperdiploidy [154]. Nevertheless, the analysis did not reveal any statistically significant correlation between the expression levels of PRINS and OS [154]. The expression dysregulation and functional role of various lncRNAs identified in MM are summarized in Table 2.

## 5. Challenges in the Delivery of ncRNA-Based Therapies in MM

The ncRNAs are emerging therapeutic tools in MM, but several challenges hamper the translation into clinical practice. The primary concern revolves around target specificity, effective delivery, and stability while avoiding “on-target” and “off-target” side effects. Currently, the focus of ncRNA application in MM is the precise targeting of MM cells while preserving healthy cells. The literature has since confirmed the regulatory roles of ncRNAs, but the off-target effects may result in undesirable outcomes [157]. Furthermore, ncRNAs undergo rapid degradation in the human body [158]. Successful treatment delivery and optimal efficacy would entail chemical modifications or encapsulation within delivery vehicles, such as nanoparticles, to improve ncRNA stability and prolong the half-life. The vehicles must effectively navigate through the bloodstream, penetrate tumor tissues, and accurately deliver the cargo exclusively within MM cells [159].

Another key measure is to ensure minimal toxicity and immunogenicity. The ncRNAs are known to elicit immune responses within the human body, which may compromise efficacy and cause adverse effects. Clinicians are now facing challenges in determining the optimal dose, frequency, and route of administration for ncRNA-based therapies. These factors can potentially influence the effectiveness and safety of the therapy greatly. Furthermore, MM cells can potentially acquire resistance towards ncRNA-based therapies as the treatment progresses, similar to other cancers. Therefore, understanding the resistance mechanisms is crucial in devising effective strategies that benefit MM patients [160]. Additionally, designing robust clinical trials for ncRNA-based therapies in MM is a major hurdle, as clinicians are required to identify suitable endpoints, patient populations, and controls to ensure the safety and efficacy of the intervention [161].

One of the highest priorities before administrating ncRNA-based treatments is identifying dependable biomarkers for patient selection, monitoring treatment response, and predicting treatment outcomes. Given the complexity and heterogeneity of MM, a thorough screening and selection process is vital in identifying suitable patients for these therapeutic interventions to improve the chances of a positive outcome. Moreover, MM is often managed using combination therapies such as chemotherapy, immunotherapy, and targeted therapies; thus, integrating ncRNA-based therapies into the treatment regimen and assessing potential synergistic or antagonistic effects is challenging. 

It is imperative to strictly adhere to ethical and consent guidelines when delivering ncRNA-based therapies in MM. The field of genetic therapies presents ethical considerations, encompassing matters such as informed consent, privacy, and the potential unanticipated consequences associated with altering gene expression by ncRNAs. It is critical to foster collaboration among researchers, clinicians, regulatory agencies, and pharmaceutical companies to address these hurdles. Ongoing progress in RNA biology, delivery technologies, and the comprehension of MM are keys to developing and integrating effective ncRNA-based therapies for MM.

## 6. Conclusions

Over the past decade, advances in therapeutics have offered a glimmer of hope for MM patients, but the condition remains largely incurable in most cases. Earlier studies have highlighted that ncRNA dysregulation is comparable to protein-coding genes in the development and progression of malignant tumors in humans, including MM. Evidence concerning the roles of ncRNAs in the onset and progression of MM implies clinical importance in the disease’s early diagnosis, prognosis, and potential therapeutic targeting. The presence of ncRNAs in the plasma of MM patients has been linked to patient prognosis, while several ncRNAs modulate important signaling pathways in MM cells, indicating the potential of becoming diagnostic and prognostic biomarkers for this disease. Nonetheless, no ncRNA has been clinically proven as a predictive indicator of MM. Therefore, further investigation should strive to understand the extent of dysregulation of these ncRNAs to enhance RNA-based therapeutics, which could either restore or suppress the expression of abnormally expressed ncRNAs. These efforts will benefit MM patients by utilizing secure, effective, and tailored ncRNAs therapy.

## Figures and Tables

**Table 1:** miRNA dysregulation in MM.

Molecular Mechanism	miRNA	Expression Dysregulation/ Direct Target		Functional Role of Dysregulation	Type of Models	Ref.
**Regulatory of BM microenvironment**	miR-202	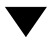	BAFF	MM cell proliferation and adhesion	MM cell	[53]
		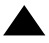	NA	Significant correlation between the β2M and κ light chain	MM patients	[54]
	miR-223	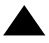	BRD4	Hematopoietic stem cells, erythroid cells, and granulocyte-monocyte progenitors development stages	Mice	[58]
	miR-485-5p	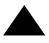	DLK1-DIO3	Decrease in cell cycle progression during the S phase and an arrest in the G1/G0 phase	MM cell	[59]
	miR-519d	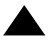	NA	Cell cycle progression and proliferation	MM cell	[62]
	miR-21	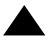	PTEN, PIAS3	Improve cell survival and enhance clonogenic development of MM cells in stromal-free circumstances	MM cell lines and mice	[55]
	miRNA-29b	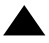	FOXP1, STAT3, NF-κB, cytokine/chemokine signaling networks	Inhibit osteoclast differentiation and suppress osteoclast activation	Osteoclast cell	[64]
	miR-138	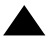	ROCK2, TRPS1, and SULF2	Inhibit bone growth	MM cell	[66]
	miR-214-3p miR-27b-3p	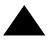	MCL1	Reduce apoptosis, develop resistance to the apoptosis mechanism, enhance myeloma fibroblasts proliferation	MM cell	[71]
	miR-146a	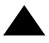	CXCL1, IL6, IL-8, IP-10, MCP-1, and CCL-5	Increase MM cell viability and migration rate	MM cell	[71]
	miR-152	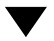	DKK1	Reduced bone mineralization	MM cell lines and MM cell	[72]
**Genomic instability**	miR-137	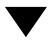	AURKA	Higher risk IgH translocations (4;14).	MM patients and MM cell lines	[73]
	miR-150 miR-22	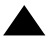	RAD54L CCNA2, LIG3	Suppress nonhomologous end-joining (ALT-NHEJ) repairs DNA double-strand breaks improve genomic instability	Natural killer cells	[86]
**Immune response**	miR-125b	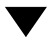	IRF4	Downregulate IRF4, c-Myc, caspase-10, and cFlip expression	MM patients and MM cell lines	[80]
	miR-29b	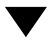	IFN	Regulate the differentiation of Th1 cells	MM cell lines, MM cell and mice	[81]
	miR-192	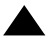	IL-17	Suppressed by IL-17 signaling cascade	MM cell lines and MM cell	[83]
	miR-10b	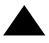	MICB	Inhibit MICB expression decline cell-mediated lysis	Natural killer cells	[86]
	miR-155 miR-221	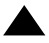	IL-6 and TNF	Stimulate IL-12 activity in DC, induce pro-inflammatory cytokines IL-6 and TNF	Myeloid DCs	[118]
	miR-22	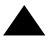	p38	Suppress IL-6, IL-23, and Th-17 response	DCs	[90]
	miR-21 miR-155	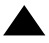	SHIP-1 and PTEN, SOCS1, BCL6, and IL13RA	Produce a higher number of MDSCs,	MM cell,	[91]
				Increase macrophage activation and trigger the pro-inflammatory response	macrophages	[93,94,95]
**Therapy resistant**	miR-15a-5p miR-17-5p miR-20a-5p miR-16-5p miR-27a	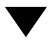	CDK5	Bortezomib-resistant	MM patient	[98]
	miR-202	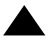	JNK/SAPK signaling pathway	Dexamethasone-sensitivity	MM cell lines and MM cell	[103]
	miR-221/222 miR-193a miR-125b	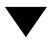	ATG12/p27-mTOR lncRNA NEAT1 p53 and SIRT1	Dexamethasone-sensitivity	MM cell lines and MM cell	[104,106]
	miR-137 miR-197	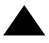	MCL-1	Dexamethasone-sensitivity	MM cell lines and MM cell	[107]
	miR-221/222 miR-152	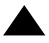	ATG12/p27-mTOR pathway,	Melphalan-resistant	MM cell lines and mice	[108]
	miR140-5p	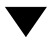	VEGFA	Inferior autophagy mechanism	MM cell	[109]
	miR-203	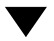	RECQ1	Protect MM cells from cytotoxicity elicited by bortezomib and melphalan	MM cell lines and MM cell	[111]
	miR-451	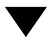	TSC1	Increase sensitivity to bortezomib, melphalan, and ascorbic acid	MM cell lines and MM cell	[112]
	miR-152	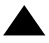	PARP	Promoting apoptosis	MM cell lines and MM cell	[72]
**Disease prognosis**	miR-17 miR-17 miR-886-5p	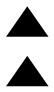	NA	Shorter OS	MM cell	[113]
	miR-15a	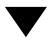	NA	Poor PFS and OS	MM cell	[114]
	miR-410	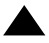	NA	Reduce survival	MM cell lines and MM cell	[115]
	miR-19a	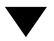	NA	Poor PFS and OS	MM patient	[116]
	miR-153 miR-296 miR-490 miR-455 miR-500 miR-642	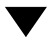	NA	Event-free survival	MM cell lines and MM cell	[117]
	miR-373 miR-548d miR-554 miR-888	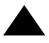	NA			[117]

BAFF, B-cell activating factor; MM, Multiple myeloma; β2M, Beta-2 microglobulin; BRD4, bromodomain containing 4; DLK1-DIO3, delta-like homolog 1 gene and the type III iodothyronine deiodinase gene; S phase, Synthesis phase; PTEN, phosphatase and tensin homolog; PIAS3, protein inhibitor of activated signal transducer and activator of transcription 3; FOXP1, forkhead box P1; STAT3, signal transducer and activator of transcription 3; NF-κB, nuclear factor-κB; ROCK2, Rho Associated Coiled-Coil Containing Protein Kinase 2; TRPS1, Transcriptional Repressor GATA Binding 1; SULF2, Sulfatase; MCL1, myeloid cell leukemia-1; CXCL1, CXC Motif Chemokine Ligand 1; IL-6, Interleukin 6; IL-8, Interleukin 8; IP-10, Interferon gamma-induced protein 10; MCP-1, monocyte chemoattractant protein-1; CCL-5, C-C Motif Chemokine Ligand 5; DKK1, Dickkopf-1; AURKA, Aurora kinase A; RAD54L, RAD54 Like; CCNA2, Cyclin A2; LIG3, DNA ligase III; ALT-NHEJ, alternative nonhomologous end-joining; DNA, Deoxyribonucleic acid; IRF4, Interferon regulatory factor 4; c-Myc, cellular Myc; cFlip, cellular FLICE-like inhibitory protein; IFN, Interferon; IL-17, Interleukin 17; MICB, major histocompatibility complex class I polypeptide-related sequence B; TNF, Tumor necrosis factor; IL-12, Interleukin 12; DC, dendritic cells; IL-23, Interleukin 23; SHIP-1, SH-2 containing inosi’ol 5′ polyphosphatase 1; SOCS1, suppressor of cytokine signaling 1; BCL6, BCL6 transcription repressor; IL13RA, interleukin 13 receptor subunit alpha 1; MDSCs, myeloid-derived suppressor cells; CDK5, cyclin-dependent kinase 5; JNK/SAPK, Jun amino-terminal kinases/Stress-activated protein kinases; lncRNA, long non coding RNA; NEAT1, nuclear paraspeckle assembly transcript 1; p53, tumor protein p53; VEGFA, vascular endothelial growth factor A; RECQ1, RecQ-like helicase 1; TSC1, tuberous sclerosis 1; PARP, poly(ADP-ribose) polymerase; OS, overall survival; PFS, progression-free survival; NA, not applicable.

**Table 2:** lncRNA transcriptional dysregulation in MM.

lncRNA	Mode of Action	Direct Target	Functional Role of Dysregulation	Types of Model	Ref.
***Oncogenes***					
**MSTRG.155519** **MSTRG.13132**	NA	CEACAM1) and FAM46C	Novel lncRNA	MM patients	[122]
**UCA1**	Decoys	CREB regulation	Increase regulation of cell cycle	Primary MM cells and cell lines	[125]
**NEAT1**	Scaffolds	miR-193a/MCL1 pathway	Increase resistance to dexamethasone; aggressive disease phase	MM cell lines	[105]
**MALAT1**	Scaffolds, decoys	miR-291/b-1	Mediates normal plasma cells transformation to MM	MM cell lines and murine	[123,132]
**PVT1**	Scaffolds, decoys, guides, or signal	miR-203a	Increase proliferation and reduce apoptosis	Primary MM cells and MM cells	
**RP4-803** **RP1-43E13.2** **ZFY-AS1** **RP11-553 L6.5**	Decoy	unknown	MM onset and progression, genetic and epigenetic modifications in MM, classify MM patients into high-risk and low-risk categories OS	MM patients	[135]
STAiR1 STAiR6 STAiR18	Scaffolds	JAK-STAT3, PI3K/Akt/mTOR, and NF-κB pathways	Promotes chromatin silencing by engaging with H3K27me3, suppress transcription, regulate heterochromatin, and epigenetic aberrations	MM cell lines	[138]
Linc00515	Decoy	ATG14	Resistance to the melphalan, MM cell autophagy	MM cell lines	[109]
**FEZF1-AS1**	Decoy	miR-610/Akt3	Cell proliferation, poor prognosis	Primary MM cells and MM cell lines	[141]
**CCAT1**	Decoy	miR-181a-5p	MM progression and development, reduce OS rate	Primary MM cells and MM cell lines	[143]
**OIP5-AS1**	Decoy	miR-410	Cell proliferation, cellular activities	Primary MM cells and MM cell lines	[115]
**CRNDE**	Scaffolds	miR-451	Disease progression, short OS	MM patients and MM cell lines	[144]
**TUG1**	Scaffolds, decoys	miR-34a-5p	Poor prognosis, disrupts cell proliferation, apoptosis, and cell cycle pathways	MM patients and MM cell lines	[146,147]
**HOTAIR**	Guide, scaffolds	JAK2/STAT3 signaling pathway	Promote chemoresistance to dexamethasone, increase cell survival and apoptotic rate	MM patients and MM cell lines	[148]
**PCAT-1**	Decoys	miR-129	Prolong MM cell survival cell proliferation, rescuing the cell cycle at the S phase, and apoptotic	MM patients and MM cell lines	[149,155,156]
**H19**	Decoy	miR-29b-3p	Early diagnosis, clinical staging, defining the patient’s severity, bortezomib resistance	MM patients and MM cell lines	[149,152]
PRINS	Decoys	G1P3	Chromosomal aberrations gain(1)(q21), del(13)(q14), del(17)(p13), t(4;14), and hyperdiploidy	MM patients	[154]
***Tumor suppressor***					
**DLEU2**	Decoys	G1 cyclins E1 and D1	Inhibits cell proliferation, differentiation, and apoptosis	MM patients	[124]
**KIAA0495**	NA	NA	Distinguish normal plasma cell from benign MGUS to symptomatic myeloma, haploinsufficiency and chromosome deletion in MM	MM patients and MM cells	[130]
STAiR2	Scaffold	Unknown novel transcripts	Inhibits DCC’s activity by alternative splicing	MM cell lines	[46,138]
**MEG3**	Decoy	miR-181a, homeobox gene A11 (HOXA11),	Suppress p53 and osteogenic differentiation of mesenchymal stem cells (MSC), compete with miR-181a to prevent the growth of tumors	MM cell lines	[123,139]
**PRAL**	Decoy	miR210	Increase sensitivity to bortezomib, suppress cell proliferation, and increase apoptotic rates, inhibit BMP2	Primary MM cells and cell lines	[142]
***Unknown***					
**STAiR15**	NA	NA	Elevate expression in nucleus MM cell disease		[46,138]

TLRs, LncRNAs, long non-coding RNAs; PVT1, plasmacytoma variant translocation 1; UCA1, urothelial cancer associated 1; MALAT1, metastasis-associated lung adenocarcinoma; ZFY-AS1, ZFY Antisense RNA 1; linc00515, long intergenic non-protein coding RNA 515; FEZF1-AS1, FEZF1 Antisense RNA 1; CCAT1, colon cancer associated transcript 1; OIP5-AS1, Opa interacting protein 5-antisense RNA 1; PCAT-1, Prostate Cancer-Associated Transcript 1; PRINS, psoriasis susceptibility-related RNA gene induced by stress; DLEU2, deletion in leukemia 2; MEG3, maternally expressed gene 3; PRAL, P53 Regulation Associated LncRNA; HOTAIR, HOX antisense intergenic RNA; NEAT1, nuclear paraspeckle assembly transcript 1; STAIR18, STAT3-induced lncRNAs 18; STAiR1, STAT3-induced lncRNAs 1; STAiR6, STAT3-induced lncRNAs 6; STAiR2, STAT3-induced lncRNAs 2; STAiR15, STAT3-induced lncRNAs 15; CRNDE, colorectal neoplasia differentially expressed; TUG1, taurine upregulated 1. CEACAM1, carcinoembryonic antigen-related cell adhesion molecule 1; FAM46C, terminal nucleotidyltransferase 5C; lncRNA, long-non coding RNA; CREB, cAMP Response Element-Binding Protein; MCL1, myeloid cell leukemia-1; MM, Multiple myeloma; OS, overall survival; JAK-STAT3, Janus kinase 2/signal transducer and activator of transcription 3; PI3K/Akt/mTOR, phosphoinositide 3-kinase/protein kinase B/mammalian target of rapamycin; NF-κB, Nuclear factor kappa B; H3K27me3, epigenetic modification to the DNA packaging protein Histone H3; ATG14, Autophagy Related 14; S phase, Synthesis Phase; G1P3, interferon inducible gene 6-16; NA, not applicable; DCC, deleted in colorectal cancer; HOXA11, homeobox gene A11; p53, tumor protein p53; MSC, mesenchymal stem cells; BMP2, bone morphogenetic protein 2 expression; MM, multiple myeloma. Note: for more information, please see the text.

## Data Availability

Data are contained within the article.

## References

[1] Li L., Wang L. (2019). Multiple Myeloma: What Do We Do About Immunodeficiency?. J. Cancer.

[2] Bird S.A., Boyd K. (2019). Multiple myeloma: An overview of management. Palliat. Care Soc. Pract..

[3] Gerecke C., Fuhrmann S., Strifler S., Schmidt-Hieber M., Einsele H., Knop S. (2016). The Diagnosis and Treatment of Multiple Myeloma. Dtsch. Arztebl. Int..

[4] Zhong L., Hao P., Zhang Q., Jiang T., Li H., Xiao J., Li C., Luo L., Xie C., Hu J. (2022). Revised International Staging System (R-ISS) stage-dependent analysis uncovers oncogenes and potential immunotherapeutic targets in multiple myeloma (MM). eLife.

[5] Rasche L., Kortüm K.M., Raab M.S., Weinhold N. (2019). The Impact of Tumor Heterogeneity on Diagnostics and Novel Therapeutic Strategies in Multiple Myeloma. Int. J. Mol. Sci..

[6] Awada H., Thapa B., Awada H., Dong J., Gurnari C., Hari P., Dhakal B. (2021). A Comprehensive Review of the Genomics of Multiple Myeloma: Evolutionary Trajectories, Gene Expression Profiling, and Emerging Therapeutics. Cells.

[7] Mao X.H., Zhuang J.L., Zhao D.D., Li X.Q., Du X., Hao M., Xu Y., Yan Y.T., Liu J.H., Fan H.S. (2020). IgH translocation with undefined partners is associated with superior outcome in multiple myeloma patients. Eur. J. Haematol..

[8] Bergsagel P.L., Kuehl W.M., Zhan F., Sawyer J., Barlogie B., Shaughnessy J. (2005). Cyclin D dysregulation: An early and unifying pathogenic event in multiple myeloma. Blood.

[9] Jovanović K.K., Escure G., Demonchy J., Willaume A., Van de Wyngaert Z., Farhat M., Chauvet P., Facon T., Quesnel B., Manier S. (2018). Deregulation and Targeting of TP53 Pathway in Multiple Myeloma. Front. Oncol..

[10] Ismail N.H., Mussa A., Zakaria N.A., Al-Khreisat M.J., Zahidin M.A., Ramli N.N., Mohammad S.N., Hassan R., Mohd Noor N.H., Iberahim S. (2022). The Role of Epigenetics in the Development and Progression of Multiple Myeloma. Biomedicines.

[11] Bhatt P., Kloock C., Comenzo R. (2023). Relapsed/Refractory Multiple Myeloma: A Review of Available Therapies and Clinical Scenarios Encountered in Myeloma Relapse. Curr. Oncol..

[12] Bianchi G., Munshi N.C. (2015). Pathogenesis beyond the cancer clone(s) in multiple myeloma. Blood.

[13] Hameed A., Brady J.J., Dowling P., Clynes M., O’Gorman P. (2014). Bone disease in multiple myeloma: Pathophysiology and management. Cancer Growth Metastasis.

[14] Zembower T.R. (2014). Epidemiology of infections in cancer patients. Cancer Treat. Res..

[15] Garrison L.P., Wang S.T., Huang H., Ba-Mancini A., Shi H., Chen K., Korves C., Dhawan R., Cakana A., van de Velde H. (2013). The cost-effectiveness of initial treatment of multiple myeloma in the U.S. with bortezomib plus melphalan and prednisone versus thalidomide plus melphalan and prednisone or lenalidomide plus melphalan and prednisone with continuous lenalidomide maintenance treatment. Oncologist.

[16] Fishilevich S., Nudel R., Rappaport N., Hadar R., Plaschkes I., Iny Stein T., Rosen N., Kohn A., Twik M., Safran M. (2017). GeneHancer: Genome-wide integration of enhancers and target genes in GeneCards. Database.

[17] Dozmorov M.G., Giles C.B., Koelsch K.A., Wren J.D. (2013). Systematic classification of non-coding RNAs by epigenomic similarity. BMC Bioinform..

[18] Leng S., Qu H., Lv X., Liu X. (2022). Role of ncRNA in multiple myeloma. Biomark. Med..

[19] Carrasco-Leon A., Ezponda T., Meydan C., Valcárcel L.V., Ordoñez R., Kulis M., Garate L., Miranda E., Segura V., Guruceaga E. (2021). Characterization of complete lncRNAs transcriptome reveals the functional and clinical impact of lncRNAs in multiple myeloma. Leukemia.

[20] Grillone K., Riillo C., Scionti F., Rocca R., Tradigo G., Guzzi P.H., Alcaro S., Di Martino M.T., Tagliaferri P., Tassone P. (2020). Non-coding RNAs in cancer: Platforms and strategies for investigating the genomic “dark matter”. J. Exp. Clin. Cancer Res..

[21] Butova R., Vychytilova-Faltejskova P., Souckova A., Sevcikova S., Hajek R. (2019). Long Non-Coding RNAs in Multiple Myeloma. Non-Coding RNA.

[22] Raimondi L., De Luca A., Giavaresi G., Raimondo S., Gallo A., Taiana E., Alessandro R., Rossi M., Neri A., Viglietto G. (2020). Non-Coding RNAs in Multiple Myeloma Bone Disease Pathophysiology. Non-Coding RNA.

[23] Beermann J., Piccoli M.T., Viereck J., Thum T. (2016). Non-coding RNAs in Development and Disease: Background, Mechanisms, and Therapeutic Approaches. Physiol. Rev..

[24] Cech T.R., Steitz J.A. (2014). The noncoding RNA revolution-trashing old rules to forge new ones. Cell.

[25] Pavet V., Portal M.M., Moulin J.C., Herbrecht R., Gronemeyer H. (2011). Towards novel paradigms for cancer therapy. Oncogene.

[26] Ponjavic J., Ponting C.P., Lunter G. (2007). Functionality or transcriptional noise? Evidence for selection within long noncoding RNAs. Genome Res..

[27] Ha M., Kim V.N. (2014). Regulation of microRNA biogenesis. Nat. Rev. Mol. Cell Biol..

[28] Broughton J.P., Lovci M.T., Huang J.L., Yeo G.W., Pasquinelli A.E. (2016). Pairing beyond the Seed Supports MicroRNA Targeting Specificity. Mol. Cell.

[29] Peschansky V.J., Wahlestedt C. (2014). Non-coding RNAs as direct and indirect modulators of epigenetic regulation. Epigenetics.

[30] Makarova J.A., Shkurnikov M.U., Wicklein D., Lange T., Samatov T.R., Turchinovich A.A., Tonevitsky A.G. (2016). Intracellular and extracellular microRNA: An update on localization and biological role. Prog. Histochem. Cytochem..

[31] Tüfekci K.U., Oner M.G., Meuwissen R.L., Genç S. (2014). The role of microRNAs in human diseases. Methods Mol. Biol..

[32] Han Z., Rosen S.T., Querfeld C. (2020). Targeting microRNA in hematologic malignancies. Curr. Opin. Oncol..

[33] Calin G.A., Croce C.M. (2006). MicroRNA signatures in human cancers. Nat. Rev. Cancer.

[34] Huang W. (2017). MicroRNAs: Biomarkers, Diagnostics, and Therapeutics. Methods Mol. Biol..

[35] O’Brien J., Hayder H., Zayed Y., Peng C. (2018). Overview of MicroRNA Biogenesis, Mechanisms of Actions, and Circulation. Front. Endocrinol..

[36] Iyer M.K., Niknafs Y.S., Malik R., Singhal U., Sahu A., Hosono Y., Barrette T.R., Prensner J.R., Evans J.R., Zhao S. (2015). The landscape of long noncoding RNAs in the human transcriptome. Nat. Genet..

[37] Uszczynska-Ratajczak B., Lagarde J., Frankish A., Guigó R., Johnson R. (2018). Towards a complete map of the human long non-coding RNA transcriptome. Nat. Rev. Genet..

[38] Fang S., Zhang L., Guo J., Niu Y., Wu Y., Li H., Zhao L., Li X., Teng X., Sun X. (2018). NONCODEV5: A comprehensive annotation database for long non-coding RNAs. Nucleic Acids Res..

[39] Camacho C.V., Choudhari R., Gadad S.S. (2018). Long noncoding RNAs and cancer, an overview. Steroids.

[40] Prensner J.R., Iyer M.K., Balbin O.A., Dhanasekaran S.M., Cao Q., Brenner J.C., Laxman B., Asangani I.A., Grasso C.S., Kominsky H.D. (2011). Transcriptome sequencing across a prostate cancer cohort identifies PCAT-1, an unannotated lincRNA implicated in disease progression. Nat. Biotechnol..

[41] Graham L.D., Pedersen S.K., Brown G.S., Ho T., Kassir Z., Moynihan A.T., Vizgoft E.K., Dunne R., Pimlott L., Young G.P. (2011). Colorectal Neoplasia Differentially Expressed (CRNDE), a Novel Gene with Elevated Expression in Colorectal Adenomas and Adenocarcinomas. Genes. Cancer.

[42] Jadaliha M., Zong X., Malakar P., Ray T., Singh D.K., Freier S.M., Jensen T., Prasanth S.G., Karni R., Ray P.S. (2016). Functional and prognostic significance of long non-coding RNA MALAT1 as a metastasis driver in ER negative lymph node negative breast cancer. Oncotarget.

[43] Gupta R.A., Shah N., Wang K.C., Kim J., Horlings H.M., Wong D.J., Tsai M.C., Hung T., Argani P., Rinn J.L. (2010). Long non-coding RNA HOTAIR reprograms chromatin state to promote cancer metastasis. Nature.

[44] Bae K., Lee M., Yoon D., Kim Y.-H., Yoon K.-A. (2017). Abstract 3137: Breast cancer anti-estrogen resistance 4 (BCAR4) is a novel oncogene in lung cancer. Cancer Res..

[45] Statello L., Guo C.J., Chen L.L., Huarte M. (2021). Gene regulation by long non-coding RNAs and its biological functions. Nat. Rev. Mol. Cell Biol..

[46] Cabili M.N., Dunagin M.C., McClanahan P.D., Biaesch A., Padovan-Merhar O., Regev A., Rinn J.L., Raj A. (2015). Localization and abundance analysis of human lncRNAs at single-cell and single-molecule resolution. Genome Biol..

[47] Chen L.L. (2016). Linking Long Noncoding RNA Localization and Function. Trends Biochem. Sci..

[48] Wang K.C., Chang H.Y. (2011). Molecular mechanisms of long noncoding RNAs. Mol. Cell.

[49] Balas M.M., Johnson A.M. (2018). Exploring the mechanisms behind long noncoding RNAs and cancer. Noncoding RNA Res..

[50] Bolha L., Ravnik-Glavač M., Glavač D. (2017). Long Noncoding RNAs as Biomarkers in Cancer. Dis. Markers.

[51] Maruyama R., Suzuki H. (2012). Long noncoding RNA involvement in cancer. BMB Rep..

[52] Ahmed E.A., Rajendran P., Scherthan H. (2022). The microRNA-202 as a Diagnostic Biomarker and a Potential Tumor Suppressor. Int. J. Mol. Sci..

[53] Shen X., Guo Y., Yu J., Qi J., Shi W., Wu X., Ni H., Ju S. (2016). miRNA-202 in bone marrow stromal cells affects the growth and adhesion of multiple myeloma cells by regulating B cell-activating factor. Clin. Exp. Med..

[54] Yu J., Qiu X., Shen X., Shi W., Wu X., Gu G., Zhu B., Ju S. (2013). miR-202 expression concentration and its clinical significance in the serum of multiple myeloma patients. Ann. Clin. Biochem..

[55] Leone E., Morelli E., Di Martino M.T., Amodio N., Foresta U., Gullà A., Rossi M., Neri A., Giordano A., Munshi N.C. (2013). Targeting miR-21 inhibits in vitro and in vivo multiple myeloma cell growth. Clin. Cancer Res..

[56] Xiong Q., Zhong Q., Zhang J., Yang M., Li C., Zheng P., Bi L.J., Ge F. (2012). Identification of novel miR-21 target proteins in multiple myeloma cells by quantitative proteomics. J. Proteome Res..

[57] Papanota A.M., Karousi P., Kontos C.K., Artemaki P.I., Liacos C.I., Papadimitriou M.A., Bagratuni T., Eleutherakis-Papaiakovou E., Malandrakis P., Ntanasis-Stathopoulos I. (2021). A Cancer-Related microRNA Signature Shows Biomarker Utility in Multiple Myeloma. Int. J. Mol. Sci..

[58] Johnnidis J.B., Harris M.H., Wheeler R.T., Stehling-Sun S., Lam M.H., Kirak O., Brummelkamp T.R., Fleming M.D., Camargo F.D. (2008). Regulation of progenitor cell proliferation and granulocyte function by microRNA-223. Nature.

[59] Mikulski D., Nowicki M., Dróźdż I., Misiewicz M., Kościelny K.P., Okoński K., Krawiec K., Perdas E., Wierzbowska A., Fendler W. (2023). High serum miR-223-3p expression level predicts complete response and prolonged overall survival in multiple myeloma patients undergoing autologous hematopoietic stem cell transplantation. Front. Oncol..

[60] Wang W., Corrigan-Cummins M., Barber E.A., Saleh L.M., Zingone A., Ghafoor A., Costello R., Zhang Y., Kurlander R.J., Korde N. (2015). Aberrant Levels of miRNAs in Bone Marrow Microenvironment and Peripheral Blood of Myeloma Patients and Disease Progression. J. Mol. Diagn..

[61] Liu D., Wang Y., Li H., Peng S., Tan H., Huang Z. (2022). Circular RNA circ-CCT3 promotes bortezomib resistance in multiple myeloma via modulating miR-223-3p/BRD4 axis. Anticancer Drugs.

[62] Berenstein R., Blau O., Nogai A., Waechter M., Slonova E., Schmidt-Hieber M., Kunitz A., Pezzutto A., Doerken B., Blau I.W. (2015). Multiple myeloma cells alter the senescence phenotype of bone marrow mesenchymal stromal cells under participation of the DLK1-DIO3 genomic region. BMC Cancer.

[63] Sun C.Y., She X.M., Qin Y., Chu Z.B., Chen L., Ai L.S., Zhang L., Hu Y. (2013). miR-15a and miR-16 affect the angiogenesis of multiple myeloma by targeting VEGF. Carcinogenesis.

[64] Rossi M., Pitari M.R., Amodio N., Di Martino M.T., Conforti F., Leone E., Botta C., Paolino F.M., Del Giudice T., Iuliano E. (2013). miR-29b negatively regulates human osteoclastic cell differentiation and function: Implications for the treatment of multiple myeloma-related bone disease. J. Cell Physiol..

[65] Wang H., Ding Q., Wang M., Guo M., Zhao Q. (2019). miR-29b inhibits the progression of multiple myeloma through downregulating FOXP1. Hematology.

[66] Botta C., Cucè M., Pitari M.R., Caracciolo D., Gullà A., Morelli E., Riillo C., Biamonte L., Gallo Cantafio M.E., Prabhala R. (2018). MiR-29b antagonizes the pro-inflammatory tumor-promoting activity of multiple myeloma-educated dendritic cells. Leukemia.

[67] Tsukamoto S., Løvendorf M.B., Park J., Salem K.Z., Reagan M.R., Manier S., Zavidij O., Rahmat M., Huynh D., Takagi S. (2018). Inhibition of microRNA-138 enhances bone formation in multiple myeloma bone marrow niche. Leukemia.

[68] Papanota A.M., Karousi P., Kontos C.K., Ntanasis-Stathopoulos I., Scorilas A., Terpos E. (2021). Multiple Myeloma Bone Disease: Implication of MicroRNAs in Its Molecular Background. Int. J. Mol. Sci..

[69] Manier S., Liu C.-J., Avet-Loiseau H., Park J., Shi J., Campigotto F., Salem K.Z., Huynh D., Glavey S.V., Rivotto B. (2017). Prognostic role of circulating exosomal miRNAs in multiple myeloma. Blood.

[70] Frassanito M.A., Desantis V., Di Marzo L., Craparotta I., Beltrame L., Marchini S., Annese T., Visino F., Arciuli M., Saltarella I. (2019). Bone marrow fibroblasts overexpress miR-27b and miR-214 in step with multiple myeloma progression, dependent on tumour cell-derived exosomes. J. Pathol..

[71] De Veirman K., Wang J., Xu S., Leleu X., Himpe E., Maes K., De Bruyne E., Van Valckenborgh E., Vanderkerken K., Menu E. (2016). Induction of miR-146a by multiple myeloma cells in mesenchymal stromal cells stimulates their pro-tumoral activity. Cancer Lett..

[72] Xu Y., Chen B., George S.K., Liu B. (2015). Downregulation of MicroRNA-152 contributes to high expression of DKK1 in multiple myeloma. RNA Biol..

[73] Qin Y., Zhang S., Deng S., An G., Qin X., Li F., Xu Y., Hao M., Yang Y., Zhou W. (2017). Epigenetic silencing of miR-137 induces drug resistance and chromosomal instability by targeting AURKA in multiple myeloma. Leukemia.

[74] Yang Y., Li F., Saha M.N., Abdi J., Qiu L., Chang H. (2015). miR-137 and miR-197 Induce Apoptosis and Suppress Tumorigenicity by Targeting MCL-1 in Multiple Myeloma. Clin. Cancer Res..

[75] Muvarak N., Kelley S., Robert C., Baer M.R., Perrotti D., Gambacorti-Passerini C., Civin C., Scheibner K., Rassool F.V. (2015). c-MYC Generates Repair Errors via Increased Transcription of Alternative-NHEJ Factors, LIG3 and PARP1, in Tyrosine Kinase-Activated Leukemias. Mol. Cancer Res..

[76] Bong I.P.N., Ng C.C., Baharuddin P., Zakaria Z. (2017). MicroRNA expression patterns and target prediction in multiple myeloma development and malignancy. Genes Genom..

[77] Mjelle R., Hegre S.A., Aas P.A., Slupphaug G., Drabløs F., Sætrom P., Krokan H.E. (2015). Cell cycle regulation of human DNA repair and chromatin remodeling genes. DNA Repair..

[78] Agarwal S., van Cappellen W.A., Guénolé A., Eppink B., Linsen S.E., Meijering E., Houtsmuller A., Kanaar R., Essers J. (2011). ATP-dependent and independent functions of Rad54 in genome maintenance. J. Cell Biol..

[79] Caracciolo D., Di Martino M.T., Amodio N., Morelli E., Montesano M., Botta C., Scionti F., Talarico D., Altomare E., Gallo Cantafio M.E. (2019). miR-22 suppresses DNA ligase III addiction in multiple myeloma. Leukemia.

[80] Morelli E., Leone E., Cantafio M.E., Di Martino M.T., Amodio N., Biamonte L., Gullà A., Foresta U., Pitari M.R., Botta C. (2015). Selective targeting of IRF4 by synthetic microRNA-125b-5p mimics induces anti-multiple myeloma activity in vitro and in vivo. Leukemia.

[81] Amodio N., Di Martino M.T., Foresta U., Leone E., Lionetti M., Leotta M., Gullà A.M., Pitari M.R., Conforti F., Rossi M. (2012). miR-29b sensitizes multiple myeloma cells to bortezomib-induced apoptosis through the activation of a feedback loop with the transcription factor Sp1. Cell Death Dis..

[82] Rossi M., Altomare E., Botta C., Gallo Cantafio M.E., Sarvide S., Caracciolo D., Riillo C., Gaspari M., Taverna D., Conforti F. (2021). miR-21 antagonism abrogates Th17 tumor promoting functions in multiple myeloma. Leukemia.

[83] Sun Y., Pan J., Mao S., Jin J. (2014). IL-17/miR-192/IL-17Rs regulatory feedback loop facilitates multiple myeloma progression. PLoS ONE.

[84] Li Y., Li D., Yan Z., Qi K., Chen L., Zhang Z., Fan G., Li H., Xu K., Li Z. (2014). Potential relationship and clinical significance of miRNAs and Th17 cytokines in patients with multiple myeloma. Leuk. Res..

[85] Jasinski-Bergner S., Mandelboim O., Seliger B. (2014). The role of microRNAs in the control of innate immune response in cancer. J. Natl. Cancer Inst..

[86] Tsukerman P., Stern-Ginossar N., Gur C., Glasner A., Nachmani D., Bauman Y., Yamin R., Vitenshtein A., Stanietsky N., Bar-Mag T. (2012). MiR-10b downregulates the stress-induced cell surface molecule MICB, a critical ligand for cancer cell recognition by natural killer cells. Cancer Res..

[87] Murray M.E., Gavile C.M., Nair J.R., Koorella C., Carlson L.M., Buac D., Utley A., Chesi M., Bergsagel P.L., Boise L.H. (2014). CD28-mediated pro-survival signaling induces chemotherapeutic resistance in multiple myeloma. Blood.

[88] Pyfferoen L., Mestdagh P., Vergote K., De Cabooter N., Vandesompele J., Lambrecht B.N., Vermaelen K.Y. (2014). Lung tumours reprogram pulmonary dendritic cell immunogenicity at the microRNA level. Int. J. Cancer.

[89] Lu C., Huang X., Zhang X., Roensch K., Cao Q., Nakayama K.I., Blazar B.R., Zeng Y., Zhou X. (2011). miR-221 and miR-155 regulate human dendritic cell development, apoptosis, and IL-12 production through targeting of p27kip1, KPC1, and SOCS-1. Blood.

[90] Liang X., Liu Y., Mei S., Zhang M., Xin J., Zhang Y., Yang R. (2015). MicroRNA-22 impairs anti-tumor ability of dendritic cells by targeting p38. PLoS ONE.

[91] Li L., Zhang J., Diao W., Wang D., Wei Y., Zhang C.Y., Zen K. (2014). MicroRNA-155 and MicroRNA-21 promote the expansion of functional myeloid-derived suppressor cells. J. Immunol..

[92] Cheng P., Corzo C.A., Luetteke N., Yu B., Nagaraj S., Bui M.M., Ortiz M., Nacken W., Sorg C., Vogl T. (2008). Inhibition of dendritic cell differentiation and accumulation of myeloid-derived suppressor cells in cancer is regulated by S100A9 protein. J. Exp. Med..

[93] Chen Y., Liu W., Sun T., Huang Y., Wang Y., Deb D.K., Yoon D., Kong J., Thadhani R., Li Y.C. (2013). 1,25-Dihydroxyvitamin D promotes negative feedback regulation of TLR signaling via targeting microRNA-155-SOCS1 in macrophages. J. Immunol..

[94] Nazari-Jahantigh M., Wei Y., Noels H., Akhtar S., Zhou Z., Koenen R.R., Heyll K., Gremse F., Kiessling F., Grommes J. (2012). MicroRNA-155 promotes atherosclerosis by repressing Bcl6 in macrophages. J. Clin. Investig..

[95] Martinez-Nunez R.T., Louafi F., Sanchez-Elsner T. (2011). The interleukin 13 (IL-13) pathway in human macrophages is modulated by microRNA-155 via direct targeting of interleukin 13 receptor alpha1 (IL13Ralpha1). J. Biol. Chem..

[96] Murphy A.J., Guyre P.M., Pioli P.A. (2010). Estradiol suppresses NF-kappa B activation through coordinated regulation of let-7a and miR-125b in primary human macrophages. J. Immunol..

[97] Rossato M., Curtale G., Tamassia N., Castellucci M., Mori L., Gasperini S., Mariotti B., De Luca M., Mirolo M., Cassatella M.A. (2012). IL-10-induced microRNA-187 negatively regulates TNF-α, IL-6, and IL-12p40 production in TLR4-stimulated monocytes. Proc. Natl. Acad. Sci. USA.

[98] Zhang L., Pan L., Xiang B., Zhu H., Wu Y., Chen M., Guan P., Zou X., Valencia C.A., Dong B. (2016). Potential role of exosome-associated microRNA panels and in vivo environment to predict drug resistance for patients with multiple myeloma. Oncotarget.

[99] Ballabio E., Armesto M., Breeze C.E., Manterola L., Arestin M., Tramonti D., Hatton C.S., Lawrie C.H. (2012). Bortezomib action in multiple myeloma: microRNA-mediated synergy (and miR-27a/CDK5 driven sensitivity)?. Blood Cancer J..

[100] Gutiérrez N.C., Sarasquete M.E., Misiewicz-Krzeminska I., Delgado M., De Las Rivas J., Ticona F.V., Fermiñán E., Martín-Jiménez P., Chillón C., Risueño A. (2010). Deregulation of microRNA expression in the different genetic subtypes of multiple myeloma and correlation with gene expression profiling. Leukemia.

[101] Wuillème-Toumi S., Robillard N., Gomez P., Moreau P., Le Gouill S., Avet-Loiseau H., Harousseau J.L., Amiot M., Bataille R. (2005). Mcl-1 is overexpressed in multiple myeloma and associated with relapse and shorter survival. Leukemia.

[102] Nencioni A., Hua F., Dillon C.P., Yokoo R., Scheiermann C., Cardone M.H., Barbieri E., Rocco I., Garuti A., Wesselborg S. (2005). Evidence for a protective role of Mcl-1 in proteasome inhibitor-induced apoptosis. Blood.

[103] Shen X., Guo Y., Qi J., Shi W., Wu X., Ni H., Ju S. (2016). Study on the Association Between miRNA-202 Expression and Drug Sensitivity in Multiple Myeloma Cells. Pathol. Oncol. Res..

[104] Xu J., Su Y., Xu A., Fan F., Mu S., Chen L., Chu Z., Zhang B., Huang H., Zhang J. (2019). miR-221/222-Mediated Inhibition of Autophagy Promotes Dexamethasone Resistance in Multiple Myeloma. Mol. Ther..

[105] Wu Y., Wang H. (2018). LncRNA NEAT1 promotes dexamethasone resistance in multiple myeloma by targeting miR-193a/MCL1 pathway. J. Biochem. Mol. Toxicol..

[106] Murray M.Y., Rushworth S.A., Zaitseva L., Bowles K.M., Macewan D.J. (2013). Attenuation of dexamethasone-induced cell death in multiple myeloma is mediated by miR-125b expression. Cell Cycle.

[107] Zhang B., Ma L., Wei J., Hu J., Zhao Z., Wang Y., Chen Y., Zhao F. (2016). miR-137 Suppresses the Phosphorylation of AKT and Improves the Dexamethasone Sensitivity in Multiple Myeloma Cells Via Targeting MITF. Curr. Cancer Drug Targets.

[108] Gullà A., Di Martino M.T., Gallo Cantafio M.E., Morelli E., Amodio N., Botta C., Pitari M.R., Lio S.G., Britti D., Stamato M.A. (2016). A 13 mer LNA-i-miR-221 Inhibitor Restores Drug Sensitivity in Melphalan-Refractory Multiple Myeloma Cells. Clin. Cancer Res..

[109] Lu D., Yang C., Zhang Z., Cong Y., Xiao M. (2018). Knockdown of Linc00515 Inhibits Multiple Myeloma Autophagy and Chemoresistance by Upregulating miR-140-5p and Downregulating ATG14. Cell Physiol. Biochem..

[110] Liu M., Liu H., Zhou J., Yu Z. (2021). miR-140-5p inhibits the proliferation of multiple myeloma cells by targeting VEGFA. Mol. Med. Rep..

[111] Viziteu E., Klein B., Basbous J., Lin Y.L., Hirtz C., Gourzones C., Tiers L., Bruyer A., Vincent L., Grandmougin C. (2017). RECQ1 helicase is involved in replication stress survival and drug resistance in multiple myeloma. Leukemia.

[112] Du J., Liu S., He J., Liu X., Qu Y., Yan W., Fan J., Li R., Xi H., Fu W. (2015). MicroRNA-451 regulates stemness of side population cells via PI3K/Akt/mTOR signaling pathway in multiple myeloma. Oncotarget.

[113] Wu P., Agnelli L., Walker B.A., Todoerti K., Lionetti M., Johnson D.C., Kaiser M., Mirabella F., Wardell C., Gregory W.M. (2013). Improved risk stratification in myeloma using a microRNA-based classifier. Br. J. Haematol..

[114] Li F., Xu Y., Deng S., Li Z., Zou D., Yi S., Sui W., Hao M., Qiu L. (2015). MicroRNA-15a/16-1 cluster located at chromosome 13q14 is down-regulated but displays different expression pattern and prognostic significance in multiple myeloma. Oncotarget.

[115] Yang N., Chen J., Zhang H., Wang X., Yao H., Peng Y., Zhang W. (2017). LncRNA OIP5-AS1 loss-induced microRNA-410 accumulation regulates cell proliferation and apoptosis by targeting KLF10 via activating PTEN/PI3K/AKT pathway in multiple myeloma. Cell Death Dis..

[116] Hao M., Zang M., Wendlandt E., Xu Y., An G., Gong D., Li F., Qi F., Zhang Y., Yang Y. (2015). Low serum miR-19a expression as a novel poor prognostic indicator in multiple myeloma. Int. J. Cancer.

[117] Chi J., Ballabio E., Chen X.H., Kušec R., Taylor S., Hay D., Tramonti D., Saunders N.J., Littlewood T., Pezzella F. (2011). MicroRNA expression in multiple myeloma is associated with genetic subtype, isotype and survival. Biol. Direct.

[118] Sehgal M., Zeremski M., Talal A.H., Ginwala R., Elrod E., Grakoui A., Li Q.G., Philip R., Khan Z.K., Jain P. (2015). IFN-α-Induced Downregulation of miR-221 in Dendritic Cells: Implications for HCV Pathogenesis and Treatment. J. Interferon Cytokine Res..

[119] Ahmad I., Valverde A., Ahmad F., Naqvi A.R. (2020). Long Noncoding RNA in Myeloid and Lymphoid Cell Differentiation, Polarization and Function. Cells.

[120] Wang F.Y., Gu Z.Y., Gao C.J. (2020). Emerging role of long non-coding RNAs in normal and malignant hematopoiesis. Chin. Med. J..

[121] Xu J., Liu B., Ma S., Zhang J., Ji Y., Xu L., Zhu M., Chen S., Wu X., Wu D. (2018). Characterizing the Tumor Suppressor Role of CEACAM1 in Multiple Myeloma. Cell. Physiol. Biochem..

[122] Lu M., Wu Y., Gao W., Tian Y., Wang G., Liu A., Chen W. (2021). Novel Non-coding RNA Analysis in Multiple Myeloma Identified Through High-Throughput Sequencing. Front. Genet..

[123] Ronchetti D., Agnelli L., Taiana E., Galletti S., Manzoni M., Todoerti K., Musto P., Strozzi F., Neri A. (2016). Distinct lncRNA transcriptional fingerprints characterize progressive stages of multiple myeloma. Oncotarget.

[124] Lerner M., Harada M., Lovén J., Castro J., Davis Z., Oscier D., Henriksson M., Sangfelt O., Grandér D., Corcoran M.M. (2009). DLEU2, frequently deleted in malignancy, functions as a critical host gene of the cell cycle inhibitory microRNAs miR-15a and miR-16-1. Exp. Cell Res..

[125] Zhang Z.S., Wang J., Zhu B.Q., Ge L. (2020). Long noncoding RNA UCA1 promotes multiple myeloma cell growth by targeting TGF-β. Eur. Rev. Med. Pharmacol. Sci..

[126] Taiana E., Bandini C., Favasuli V.K., Ronchetti D., Silvestris I., Puccio N., Todoerti K., Erratico S., Giannandrea D., Bolli N. (2023). Activation of long non-coding RNA NEAT1 leads to survival advantage of multiple myeloma cells by supporting a positive regulatory loop with DNA repair proteins. Haematologica.

[127] Zhan F., Barlogie B., Arzoumanian V., Huang Y., Williams D.R., Hollmig K., Pineda-Roman M., Tricot G., van Rhee F., Zangari M. (2007). Gene-expression signature of benign monoclonal gammopathy evident in multiple myeloma is linked to good prognosis. Blood.

[128] Wong K.Y., Li Z., Zhang X., Leung G.K., Chan G.C., Chim C.S. (2015). Epigenetic silencing of a long non-coding RNA KIAA0495 in multiple myeloma. Mol. Cancer.

[129] Pang J.C., Li K.K., Lau K.M., Ng Y.L., Wong J., Chung N.Y., Li H.M., Chui Y.L., Lui V.W., Chen Z.P. (2010). KIAA0495/PDAM is frequently downregulated in oligodendroglial tumors and its knockdown by siRNA induces cisplatin resistance in glioma cells. Brain Pathol..

[130] Teoh P.J., Chung T.H., Sebastian S., Choo S.N., Yan J., Ng S.B., Fonseca R., Chng W.J. (2014). p53 haploinsufficiency and functional abnormalities in multiple myeloma. Leukemia.

[131] Amodio N., Stamato M.A., Juli G., Morelli E., Fulciniti M., Manzoni M., Taiana E., Agnelli L., Cantafio M.E.G., Romeo E. (2018). Drugging the lncRNA MALAT1 via LNA gapmeR ASO inhibits gene expression of proteasome subunits and triggers anti-multiple myeloma activity. Leukemia.

[132] Stamato M.A., Juli G., Romeo E., Ronchetti D., Arbitrio M., Caracciolo D., Neri A., Tagliaferri P., Tassone P., Amodio N. (2017). Inhibition of EZH2 triggers the tumor suppressive miR-29b network in multiple myeloma. Oncotarget.

[133] Deming S.L., Nass S.J., Dickson R.B., Trock B.J. (2000). C-myc amplification in breast cancer: A meta-analysis of its occurrence and prognostic relevance. Br. J. Cancer.

[134] Nagoshi H., Taki T., Hanamura I., Nitta M., Otsuki T., Nishida K., Okuda K., Sakamoto N., Kobayashi S., Yamamoto-Sugitani M. (2012). Frequent PVT1 rearrangement and novel chimeric genes PVT1-NBEA and PVT1-WWOX occur in multiple myeloma with 8q24 abnormality. Cancer Res..

[135] Zhou M., Zhao H., Wang Z., Cheng L., Yang L., Shi H., Yang H., Sun J. (2015). Identification and validation of potential prognostic lncRNA biomarkers for predicting survival in patients with multiple myeloma. J. Exp. Clin. Cancer Res..

[136] Yang M., Zhang L., Wang X., Zhou Y., Wu S. (2018). Down-regulation of miR-203a by lncRNA PVT1 in multiple myeloma promotes cell proliferation. Arch. Med. Sci..

[137] Chong P.S.Y., Chng W.-J., de Mel S. (2019). STAT3: A Promising Therapeutic Target in Multiple Myeloma. Cancers.

[138] Binder S., Hösler N., Riedel D., Zipfel I., Buschmann T., Kämpf C., Reiche K., Burger R., Gramatzki M., Hackermüller J. (2017). STAT3-induced long noncoding RNAs in multiple myeloma cells display different properties in cancer. Sci. Rep..

[139] Shen X., Bai H., Zhu H., Yan Q., Yang Y., Yu W., Shi Q., Wang J., Li J., Chen L. (2018). Long Non-Coding RNA MEG3 Functions as a Competing Endogenous RNA to Regulate HOXA11 Expression by Sponging miR-181a in Multiple Myeloma. Cell Physiol. Biochem..

[140] Zhuang W., Ge X., Yang S., Huang M., Zhuang W., Chen P., Zhang X., Fu J., Qu J., Li B. (2015). Upregulation of lncRNA MEG3 Promotes Osteogenic Differentiation of Mesenchymal Stem Cells From Multiple Myeloma Patients By Targeting BMP4 Transcription. Stem Cells.

[141] Li Q.Y., Chen L., Hu N., Zhao H. (2018). Long non-coding RNA FEZF1-AS1 promotes cell growth in multiple myeloma via miR-610/Akt3 axis. Biomed. Pharmacother..

[142] Xiao G., Li Y., Wang Y., Zhao B., Zou Z., Hou S., Jia X., Liu X., Yao Y., Wan J. (2018). LncRNA PRAL is closely related to clinical prognosis of multiple myeloma and the bortezomib sensitivity. Exp. Cell Res..

[143] Chen L., Hu N., Wang C., Zhao H., Gu Y. (2018). Long non-coding RNA CCAT1 promotes multiple myeloma progression by acting as a molecular sponge of miR-181a-5p to modulate HOXA1 expression. Cell Cycle.

[144] Meng Y.B., He X., Huang Y.F., Wu Q.N., Zhou Y.C., Hao D.J. (2017). Long Noncoding RNA CRNDE Promotes Multiple Myeloma Cell Growth by Suppressing miR-451. Oncol. Res..

[145] David A., Zocchi S., Talbot A., Choisy C., Ohnona A., Lion J., Cuccuini W., Soulier J., Arnulf B., Bories J.-C. (2021). The long non-coding RNA CRNDE regulates growth of multiple myeloma cells via an effect on IL6 signalling. Leukemia.

[146] Zhang Y., Zhao D., Li S., Xiao M., Zhou H., Yang S., Hao Y., Dong S. (2020). Long non-coding RNA TUG1 knockdown hinders the tumorigenesis of multiple myeloma by regulating the microRNA-34a-5p/NOTCH1 signaling pathway. Open Life Sci..

[147] Farooqi A.A., Tabassum S., Ahmad A. (2017). MicroRNA-34a: A Versatile Regulator of Myriads of Targets in Different Cancers. Int. J. Mol. Sci..

[148] Guan R., Wang W., Fu B., Pang Y., Lou Y., Li H. (2019). Increased lncRNA HOTAIR expression promotes the chemoresistance of multiple myeloma to dexamethasone by regulating cell viability and apoptosis by mediating the JAK2/STAT3 signaling pathway. Mol. Med. Rep..

[149] Shen X., Zhang Y., Wu X., Guo Y., Shi W., Qi J., Cong H., Wang X., Wu X., Ju S. (2017). Upregulated lncRNA-PCAT1 is closely related to clinical diagnosis of multiple myeloma as a predictive biomarker in serum. Cancer Biomark..

[150] Shen X., Kong S., Yang Q., Yin Q., Cong H., Wang X., Ju S. (2020). PCAT-1 promotes cell growth by sponging miR-129 via MAP3K7/NF-κB pathway in multiple myeloma. J. Cell Mol. Med..

[151] Shen X., Shen P., Yang Q., Yin Q., Wang F., Cong H., Wang X., Ju S. (2019). Knockdown of long non-coding RNA PCAT-1 inhibits myeloma cell growth and drug resistance via p38 and JNK MAPK pathways. J. Cancer.

[152] Pan Y., Zhang Y., Liu W., Huang Y., Shen X., Jing R., Pu J., Wang X., Ju S., Cong H. (2019). LncRNA H19 overexpression induces bortezomib resistance in multiple myeloma by targeting MCL-1 via miR-29b-3p. Cell Death Dis..

[153] Sun Y., Pan J., Zhang N., Wei W., Yu S., Ai L. (2017). Knockdown of long non-coding RNA H19 inhibits multiple myeloma cell growth via NF-κB pathway. Sci. Rep..

[154] Sedlarikova L., Bollova B., Radova L., Brozova L., Jarkovsky J., Almasi M., Penka M., Kuglík P., Sandecká V., Stork M. (2018). Circulating exosomal long noncoding RNA PRINS-First findings in monoclonal gammopathies. Hematol. Oncol..

[155] Hideshima T., Anderson K.C. (2021). Signaling Pathway Mediating Myeloma Cell Growth and Survival. Cancers.

[156] Liu Z., Gao H., Peng Q., Yang Y. (2020). Long Noncoding RNA LUCAT1 Promotes Multiple Myeloma Cell Growth by Regulating the TGF-β Signaling Pathway. Technol. Cancer Res. Treat..

[157] Hueso M., Mallén A., Suñé-Pou M., Aran J.M., Suñé-Negre J.M., Navarro E. (2021). ncRNAs in Therapeutics: Challenges and Limitations in Nucleic Acid-Based Drug Delivery. Int. J. Mol. Sci..

[158] Béthune J., Artus-Revel C.G., Filipowicz W. (2012). Kinetic analysis reveals successive steps leading to miRNA-mediated silencing in mammalian cells. EMBO Rep..

[159] Yusuf A., Almotairy A.R.Z., Henidi H., Alshehri O.Y., Aldughaim M.S. (2023). Nanoparticles as Drug Delivery Systems: A Review of the Implication of Nanoparticles&rsquo; Physicochemical Properties on Responses in Biological Systems. Polymers.

[160] Chen X., Mangala L.S., Rodriguez-Aguayo C., Kong X., Lopez-Berestein G., Sood A.K. (2018). RNA interference-based therapy and its delivery systems. Cancer Metastasis Rev..

[161] Chen B., Dragomir M.P., Yang C., Li Q., Horst D., Calin G.A. (2022). Targeting non-coding RNAs to overcome cancer therapy resistance. Signal Transduct. Target. Ther..

